# An Abundant Evolutionarily Conserved CSB-PiggyBac Fusion Protein Expressed in Cockayne Syndrome

**DOI:** 10.1371/journal.pgen.1000031

**Published:** 2008-03-21

**Authors:** John C. Newman, Arnold D. Bailey, Hua-Ying Fan, Thomas Pavelitz, Alan M. Weiner

**Affiliations:** 1 Department of Biochemistry, School of Medicine, University of Washington, Seattle, Washington, United States of America; 2 Fox Chase Cancer Center, Philadelphia, Pennsylvania, United States of America; Stanford University, United States of America

## Abstract

Cockayne syndrome (CS) is a devastating progeria most often caused by mutations in the *CSB* gene encoding a SWI/SNF family chromatin remodeling protein. Although all CSB mutations that cause CS are recessive, the complete absence of CSB protein does not cause CS. In addition, most CSB mutations are located beyond exon 5 and are thought to generate only C-terminally truncated protein fragments. We now show that a domesticated PiggyBac-like transposon *PGBD3*, residing within intron 5 of the *CSB* gene, functions as an alternative 3′ terminal exon. The alternatively spliced mRNA encodes a novel chimeric protein in which *CSB* exons 1–5 are joined in frame to the PiggyBac transposase. The resulting CSB-transposase fusion protein is as abundant as CSB protein itself in a variety of human cell lines, and continues to be expressed by primary CS cells in which functional CSB is lost due to mutations beyond exon 5. The CSB-transposase fusion protein has been highly conserved for at least 43 Myr since the divergence of humans and marmoset, and appears to be subject to selective pressure. The human genome contains over 600 nonautonomous PGBD3-related MER85 elements that were dispersed when the PGBD3 transposase was last active at least 37 Mya. Many of these MER85 elements are associated with genes which are involved in neuronal development, and are known to be regulated by CSB. We speculate that the CSB-transposase fusion protein has been conserved for host antitransposon defense, or to modulate gene regulation by MER85 elements, but may cause CS in the absence of functional CSB protein.

## Author Summary

For reasons that are still unclear, genetic defects in DNA repair can cause diseases that resemble aspects of premature ageing (“segmental progerias”). Cockayne syndrome (CS) is a particularly devastating progeria most commonly caused by mutations in the CSB chromatin remodeling gene. About 43 million years ago, before humans diverged from marmosets, one of the last PiggyBac transposable elements to invade the human lineage landed within intron 5 of the 21 exon CSB gene. As a result, the CSB locus now encodes two equally abundant proteins generated by alternative mRNA splicing: the original full length CSB protein, and a novel CSB-PiggyBac fusion protein in which the N-terminus of CSB is fused to the complete PiggyBac transposase. Conservation of the CSB-PiggyBac fusion protein since marmoset suggests that it is normally beneficial, demonstrating once again that “selfish” transposable elements can be exploited or “domesticated” by the host. More importantly, almost all CSB mutations that cause CS continue to make the CSB-PiggyBac fusion protein, whereas a mutation that compromises both does not cause CS. Thus the fusion protein which is beneficial in the presence of functional CSB may be harmful in its absence. This may help clarify the cause of CS and other progerias.

## Introduction

The human genome is replete with interlopers — transposable DNA elements, retrotransposable RNA elements such as SINEs and LINEs, and a dizzying variety of lesser-known elements — which together account for as much as half of our DNA [1]. Although much of this “junk” DNA is selfish and surprisingly harmless, the constant turnover of these elements is an important source of insertional mutagenesis with benign [2] and malign [3] consequences. Indeed, eukaryotes often recruit mobile elements to perform critical functions — a process known as domestication or exaptation [4]. For example, the RAG1 recombinase, which diversifies the adaptive immune response in mammals, was domesticated aeons ago from a Transib-family transposase [5]. A similarly domesticated DNA transposon is responsible for the programmed genomic rearrangements found in many ciliates [6], and a pogo-like transposase gave rise to the centromeric CEN-P protein family [7] which mediates host genome surveillance for retrotransposons in *Schizosaccharomyces pombe* [8]. More recently in the primate lineage, a *mariner*-like transposase was fused to a SET histone methyltransferase domain by *de novo* exonization; the fusion protein retains the ancestral DNA binding activity of the transposase, and may function as a transcriptional regulator at dispersed *mariner*-like repeat elements [9]. Here we report identification of an evolutionarily conserved PiggyBac transposase fusion protein that may play a critical, and previously unsuspected, role in a well-studied human disease, Cockayne syndrome (CS).

PiggyBac elements, first characterized in the cabbage looper moth *Trichoplusia ni* [10],[11], have now been identified in a variety of eukaryotes from protozoa [12] to primates [1]. A typical PiggyBac element contains a 1.8 kb ORF encoding a 68 kDa transposase; the boundaries of the element are defined by 13–15 nt terminal inverted repeats, which are in turn flanked by a duplication of the target site TTAA [13]. The *T. ni* PiggyBac transposon is a useful tool for germline manipulation because it is active in a wide range of species including mammals [14] and has been considered as a possible gene therapy vector [15]. The five PiggyBac elements in the human genome (*PGBD1-5*) are variously conserved among vertebrates; *PGBD5* dates to before the teleost/tetrapod split, whereas *PGBD3* and *PGBD4* are restricted to primates [1],[13].

CS is a devastating inherited progeria characterized by severe post-natal growth failure and progressive neurological dysfunction [16]. Most cases of CS reflect mutations in the Cockayne syndrome Group B (*CSB*, also known as *ERCC6*) gene, a SWI/SNF-like DNA-dependent ATPase [17]–[19] that can wind DNA [20] and remodel chromatin *in vitro* [21]; the remaining cases of CS are caused by mutations in the *CSA* gene, and by rare alleles of the xeroderma pigmentosum genes *XPB*, *XPD*, and *XPG* [22]. All of these factors were originally identified as being involved in the transcription-coupled repair of UV-induced DNA damage [23],[24]. While searching for an activity that could better explain the CS phenotype, we found that CSB has a general chromatin remodeling function [25] which could account for the pleiotropic effects of *CSB* mutations and the characteristic wasting of CS [26]. Alternatively, CS may be caused by defects in transcription initiation [27],[28], or by a partial failure to repair oxidative DNA damage. CSB is known to enhance repair of 8-hydroxyguanine lesions [29], and mice doubly mutant for CSB and the 8-hydroxyguanine glycosylase OGG1 are severely deficient in global repair of endogenous oxidative DNA damage [30]. Similarly, complete inactivation of nucleotide excision repair (NER) in mice doubly mutant for CSB and XPA mimics CS and suppresses the somatotroph axis [31],[32]. As yet unexplained, however, is why complete absence of CSB does not cause CS, although all CS mutations are recessive [33]–[35].

Here we show that the PiggyBac transposable element *PGBD3* embedded within intron 5 of the CSB gene functions as an alternative 3′ terminal exon (“exon trap”); as a result, alternative splicing of the CSB primary transcript generates two mRNAs, one encoding all 21 exons of the CSB protein, and the other an equally abundant CSB-related protein in which the first 5 exons of CSB are fused to the PGBD3 transposase. Sequence comparisons of *PGBD3* with PiggyBac pseudogenes in humans and other primates suggest that *PGBD3* was domesticated soon after it transposed into the *CSB* gene. Indeed, conservation of the alternatively spliced *PGBD3* element in the *CSB* genes of chimpanzee, orangutan, Rhesus macaque and marmoset over at least 43 Myr of evolution [36], together with a preponderance of synonymous mutations, strongly suggest that the fusion protein has been selected for an advantageous function in its primate host. We speculate that the CSB-transposase fusion protein originally played a role in host genome defense by repressing transposition of autonomous PGBD3 elements and the hundreds of nonautonomous PGBD3-dependent MER85 elements derived from them. We also find an association of MER85 elements with a subset of CSB-regulated genes and genes involved in neuronal development, suggesting that the fusion protein may later have acquired the ability to modulate gene regulatory networks. Finally, we show that the CSB-transposase fusion protein continues to be expressed in CS primary cells lacking functional CSB protein, implying that the fusion protein could contribute to the CS phenotype, or even transform the mild UV sensitivity caused by complete loss of CSB-related proteins [33] into a true progeria.

## Results

### CSB-PGBD3 Fusion Transcript Is a Major Product of the *CSB* Gene

**Figure 1:**
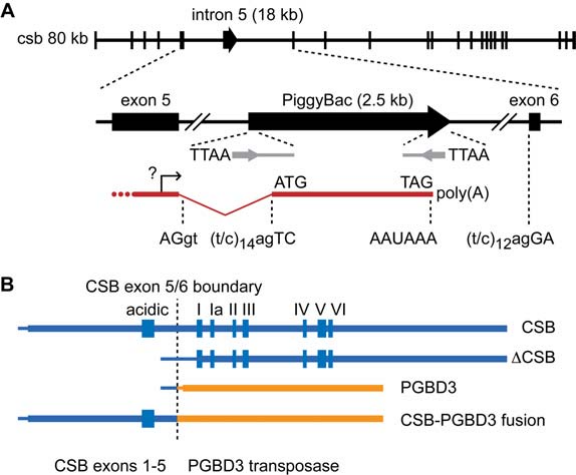
The PiggyBac Element *PGBD3* and the *CSB-PGBD3* Fusion Transcript. (A) *PGBD3* is located in intron 5 of the *CSB* gene, and appears to be independently transcribed from a cryptic promoter within *CSB* exon 5. *PGBD3* contains a 3′ splice site in frame with the 5′ splice site of *CSB* exon 5, and a polyadenylation signal (exon, upper case; intron, lower case). The element is flanked by a target site TTAA duplication just outside the left (100 nt) and right (40 nt) split ends of a MER85 repeat element (gray lines, not to scale) with 13 nt terminal inverted repeats (gray arrows, not to scale). (B) The 3.4 kb *CSB-PGBD3* fusion transcript encodes a protein of 1061 amino acids, including the 465 N-terminal residues of CSB — up to but not including the central ATPase motifs (Roman numerals) — and the entire PGBD3 transposase. Two transcripts may be generated from the cryptic promoter with exon 5: The *PGBD3* transposase, and a potential N-terminally deleted CSB transcript (ΔCSB). Absence of an early start codon suggests that translation of the latter would begin within the first ATPase motif. Thin lines denote 5′ untranslated sequence.

Intron 5 of the human *CSB* gene is host to a PiggyBac transposable element known as *PGBD3* (Figure 1A). We initially noted that the RefSeq transcript for *PGBD3* (along with four of seven deposited mRNAs) consists of the 3′ region of CSB exon 5 spliced to the entire PiggyBac coding region. The *PGBD3* transposase ORF is flanked by a 3′ splice acceptor site just 7 nt upstream of the first methionine, and a polyadenylation site about 130 nt downstream of the termination codon. Moreover, the *CSB* and *PGBD3* coding regions are in frame across this splice junction, suggesting that transcripts initiating at a normal CSB promoter could be alternatively spliced to the PiggyBac element instead of exon 6, thus generating a CSB-PGBD3 fusion protein ( 1B). In this fusion protein, the N-terminal 465 residues of CSB (including the acidic domain but not the ATPase) would be tethered to the entire PiggyBac transposase. In fact, two of the seven *PGBD3* GenBank mRNA sequences (BC034479 and AK291018) appear to be just such variants, starting at either the noncoding *CSB* exon 1 (AK291018) or an alternative noncoding exon 1 (BC034479) and ending just beyond the *PGBD3* polyadenylation site. Four other *PGBD3* GenBank mRNA sequences consist of the 3′ region of *CSB* exon 5 spliced to the entire PiggyBac coding region, suggesting the existence of an unusual cryptic promoter within exon 5 (the sixth mRNA, likely incomplete, begins within the transposase ORF). We sought to confirm the existence of such alternatively spliced transcripts, and to determine whether the transcripts initiate at the putative cryptic promoter within exon 5 or at a normal *CSB* transcription start site.

**Figure 2:**
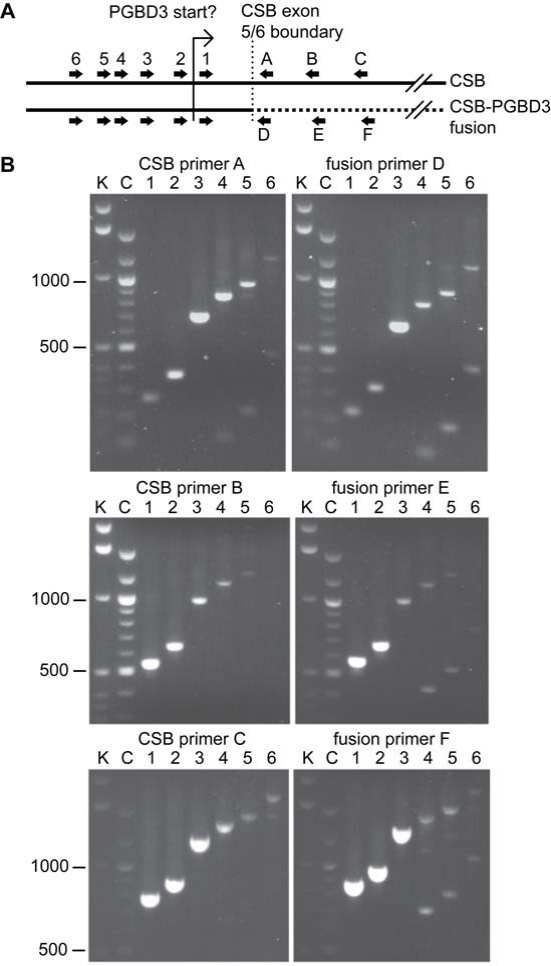
The Extent and Abundance of the *CSB-PGBD3* Fusion Transcript Assayed by Quantitative Real-Time RT-PCR. (A) Location of six upstream primer sites, common to both the *CSB* and *CSB-PGBD3* fusion transcripts; and three downstream primer sites specific to each transcript. (B) Most of the tested primer pairs generated clean PCR products of the predicted size; identity was verified by sequencing. The smaller products generated by primers A, D, E and F appear to be alternatively spliced transcripts lacking the 745 nt CSB exon 5; these transcripts are not predicted to encode a functional protein. The products of the 40-cycle real-time protocol were resolved by 1% agarose gel electrophoresis. K, Invitrogen 1 kb ladder; C, NEB 100 bp ladder; markers in bp.

We were able to detect the predicted *CSB-PGBD3* fusion transcripts by quantitative, real-time RT-PCR (Q-RT-PCR) using HeLa mRNA as template, forward primers for the 3′ half of *CSB* exon 5 which is shared by the *CSB* and predicted fusion mRNAs, and reverse primers which are specific for either *CSB* exon 6 or the *PGBD3* element ( 2A). The fusion products exhibited the expected size ( 2B) and sequence (data not shown), and were approximately 2-fold more abundant than the equivalent *CSB* products (Figure S1). Moreover, we readily detected fusion products using forward primers for *CSB* exons 2, 3 and 4, indicating that a significant fraction of the *CSB-PGBD3* fusion transcripts initiate far upstream of the putative cryptic promoter, presumably at a natural *CSB* initiation site. These full-length *CSB-PGBD3* fusion transcripts do not reflect template strand switching by reverse transcriptase or recombination during PCR [37] within exon 5, because alternatively spliced fusion transcripts lacking exon 5 were also observed (Figure 2), and the abundance of the fusion products was not diminished in control experiments where either one of the potentially recombining mRNAs was sequestered within a cDNA:mRNA hybrid by a preliminary reverse transcription step using an mRNA-specific primer (data not shown).

Using a subset of these primer combinations, we also detected *CSB-PGBD3* fusion transcripts in three other cell lines: hTERT-immortalized WI38 normal lung fibroblasts, and hTERT-immortalized CS1AN CSB fibroblasts rescued with CSB-wt cDNA (CSB-wt line) or mock-rescued with enhanced green fluorescent protein (CSB-null line) [25]. In all four lines, the fusion transcripts were more abundant than the CSB transcripts — as much as 13- to 26-fold more abundant in the immortalized WI38 line (Figure S2).

The *CSB-PGBD3* fusion transcript, apparently initiating at or near the normal *CSB* start site, appears to be the only major alternatively spliced transcript expressed from the *CSB/PGBD3* gene. First, the transposase coding region is not an alternative exon within full-length *CSB* mRNA, because combinations of two upstream primers from the PiggyBac element and four downstream primers located in *CSB* exons 6, 7, 8 and 9 failed to produce RT-PCR products in any of the four cell lines tested (data not shown). Second, the *CSB* and *CSB-PGBD3* transcripts lacking exon 5 appear to be scarce (Figure 2, compare smaller and larger bands in lanes 4–6 for CSB primer A and fusion primer D). And third, as judged by Q-RT-PCR, the 3′ region of the *CSB* mRNA appears to be less abundant than the 5′ region (data not shown), arguing that the putative cryptic promoter within *CSB* exon 5 does not generate significant quantities of an N-terminally truncated *CSB* mRNA (ΔCSB, see Figure 1B).

### Detection of the CSB-PGBD3 Fusion Protein

**Figure 3:**
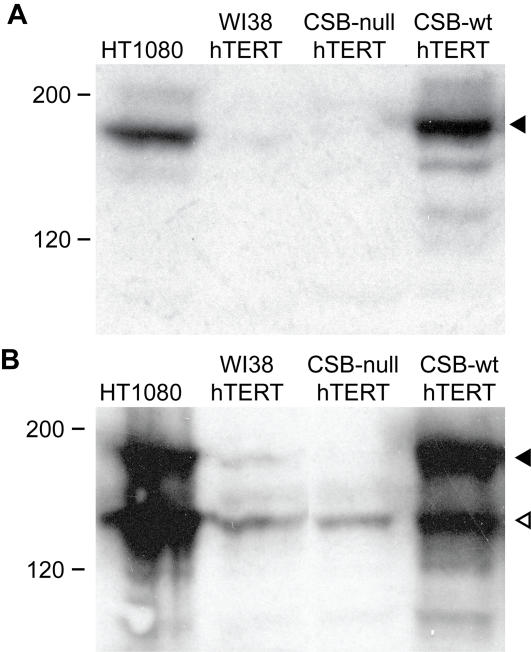
CSB-PGBD3 Fusion Protein Identified by Anti-CSB Western Blotting. (A) Western blot using a C-terminal antibody against CSB reveals the expected 170 kDa major band for full length CSB (filled arrowhead). CSB is weakly expressed in hTERT-immortalized WI38 cells, but not at all in hTERT-immortalized CS1AN cells (CSB-null) until rescue with CSB cDNA (CSB-wt). (B) Western blot using an N-terminal antibody against CSB reveals both full length CSB (filled arrowhead) and, in all cell lines, a second major band of approximately 140 kDa (hollow arrowhead) that we identify in Figure 4 as a CSB-PGBD3 fusion protein. The fusion protein is more abundant than CSB in WI38/hTERT cells, and is strongly expressed in immortalized CS1AN cells before and after rescue. CS1AN is a compound heterozygote line; one *CSB* allele contains an early truncating mutation (K337STOP) but a second allele with a 100 nt deletion in exon 13 should permit expression of the fusion protein. CSB and the CSB-PGBD3 fusion protein both migrate more slowly than predicted based on molecular mass alone, presumably due to the N-terminal acidic domain (see text). Markers in kDa.

Consistent with the Q-RT-PCR data, we detected the CSB-PGBD3 fusion protein in four different cell lines (HT1080, WI38/hTERT, CSB-null and CSB-wt) by Western blotting with antibodies specific for the N- and C-termini of CSB protein. The C-terminal antibody revealed one major band of the size expected for intact CSB protein (Figure 3A), whereas the N-terminal antibody revealed two major bands — intact CSB and a smaller band of approximately the size expected for the fusion protein (Figure 3B). Notably, the fusion band was present in an immortalized CSB-null line derived from the severely affected individual CS1AN — a compound heterozygote consisting of one *CSB* allele with an early truncating mutation (K337STOP) and a second allele with a 100 nt deletion in exon 13 [38]. The latter allele should, and does, permit normal expression of the fusion protein in this CS cell line (Figure 3B). The fusion band was also seen in the Saos-2 osteosarcoma and MRC5 fibroblast cell lines (Figure S3).

**Figure 4:**
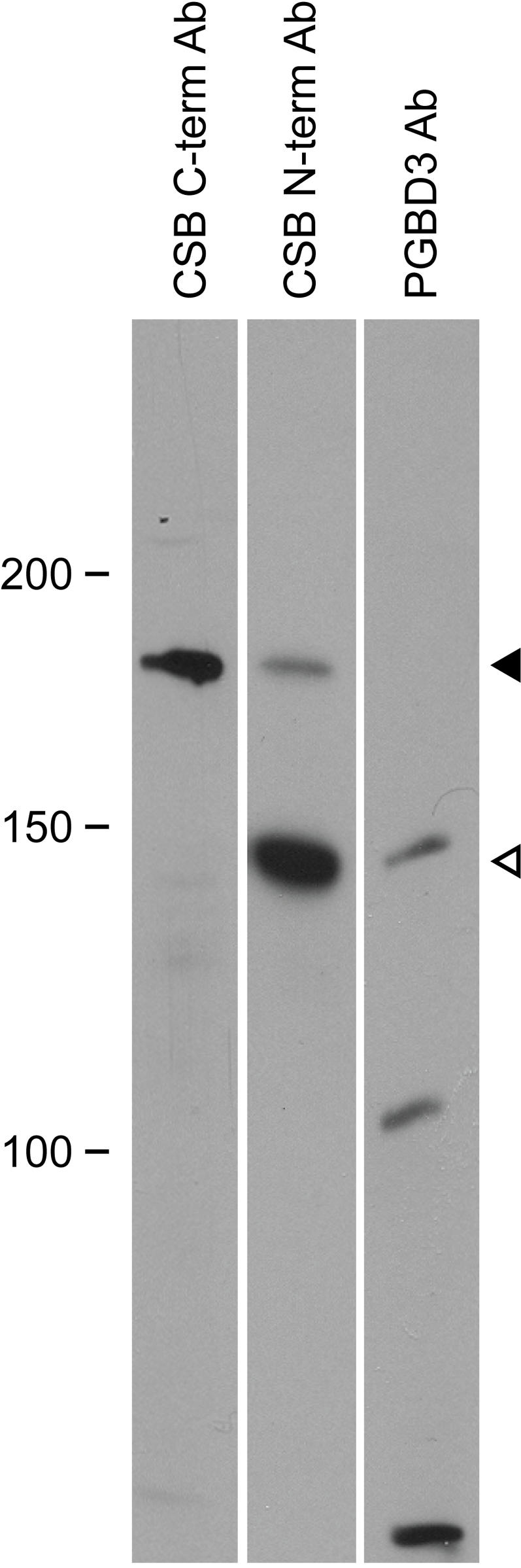
CSB-PGBD3 Fusion Protein Reacts with Anti-PGBD3 Antibody. Western blot of HT1080 extracts using CSB C-terminal and N-terminal antibodies, and a commercial antipeptide antibody to PGBD3. The PGBD3 antibody reacts with the same 140 kDa band as the N-terminal CSB antibody. CSB decreases relative to CSB-PGBD3 fusion protein as cells approach confluence; compare logarithmically growing HT1080 (Figure 3B, leftmost lane) with confluent cells (Figure 4, middle panel). The lower band on the PGBD3 panel is likely PGBD3 itself (predicted 68 kDa, presumably initiating at the cryptic promoter within CSB exon 5), while the middle 105 kDa band may reflect crossreaction with PGBD1 (predicted 93 kDa) which contains a region homologous to the peptide epitope. Markers in kDa.

To confirm the identity of the CSB-PGBD3 fusion band as visualized with the N-terminal CSB antibody (Figure 3), we used a commercial PGBD3-specific antibody. The PGBD3 antibody revealed three major bands on Western blotting, including one that comigrates with the fusion band (Figure 4). The CSB-PGBD3 fusion protein (with calculated mass 120 kDa and pI 6.15) migrates more slowly than expected, but this is commonly observed for acidic proteins [39], and the endogenous CSB-PGBD3 fusion protein comigrates with recombinant tagged CSB-PGBD3 fusion protein after correction for tag size (data not shown). In contrast, CSB has a calculated pI of 8.2 and migrates as expected for a mass of 168 kDa. We conclude that the endogenous protein reacting with both N-terminal CSB antibody (Figure 3) and the PGBD3-specific antibody (Figure 4) is the abundant CSB-PGBD3 fusion protein.

### The CSB-Transposase Fusion Protein is Expressed in Primary CS Cells

**Figure 5:**
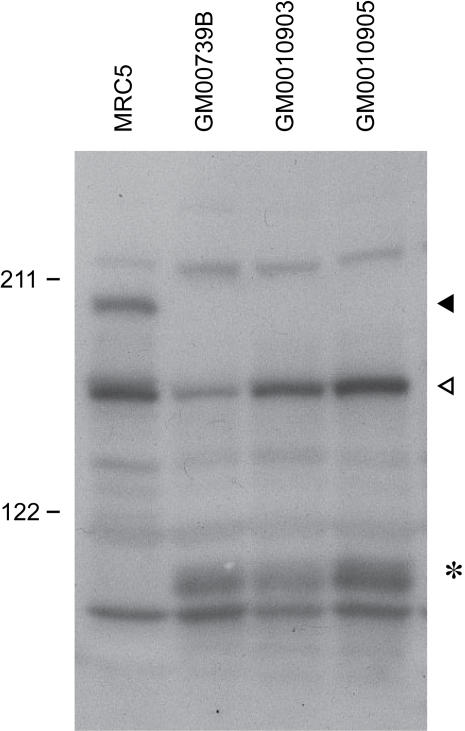
CSB-PGBD3 Fusion Protein in Primary CS Cells Lacking Intact CSB. Western blots using an N-terminal CSB antibody reveal only the fusion protein (hollow arrowhead) and not full-length CSB (filled arrowhead) in three different primary CSB cells. GM00739B is derived from patient CS1AN and is the parent of our hTERT-immortalized derivatives (CSB-wt and CSB-null lines), thus demonstrating that the fusion protein is not an artifact of immortalization. GM10903 and GM10905 are from two patients diagnosed with the DeSanctis-Cacchione variant of XP (XP-DSC; XP61SF and XP63SF in Table S1). The clinical overlap between CSB/XP-DSC and CSB/CS is substantial, including photosensitivity (but not skin cancers), mental retardation and severe growth failure [77]; in fact, the same homozygous R735STOP mutation shared by both patients is also associated with classical CS in another patient (CS1TAN). A broad unidentified band or doublet of about 100 kDa (asterisk) is seen in the primary lines (GM00739B, GM10903, and GM10905) but is absent when cells are spontaneously transformed (MRC5) or immortalized by SV40 (Figure S3) or hTERT (Figure 3).

A tabulation of all reported CS cases with known mutations in CSB reveals that 21 of 24 retain at least one allele that should allow continued expression of the CSB-transposase fusion protein (Table S1). To confirm that CS cells express the fusion protein in the absence of intact CSB, as seen for the hTERT-immortalized CS1AN line (Figure 3B), we screened three different primary CSB cells (GM10903, GM10905, and GM00739B derived from patient CS1AN) none of which, as expected, exhibited intact CSB protein. All, however, express the fusion protein (Figure 5). Nor is expression an artifact of immortalization, as the abundance of the fusion protein was similar in primary GM00739B cells (Figure 5) and derived cell lines immortalized either with hTERT (Figure 2, GM00739B) or SV40 (Figure S3, CS1AN/SV).

### The CSB-PGBD3 Fusion Protein Sequence and Splice Sites are Conserved in Primates

**Figure 6:**
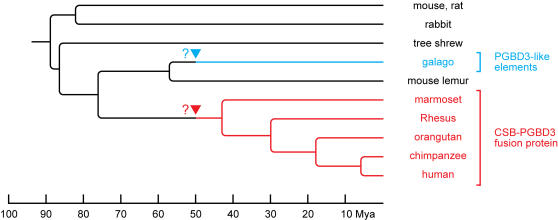
Phylogeny of *PGBD3* and the CSB-PGBD3 Fusion Protein. *PGBD3* inserted into the *CSB* gene prior to the divergence of human and marmoset approximately 43 Mya, and has been highly conserved in all members of this lineage. An element very similar to ancestral *PGBD3* also invaded the galago genome after the divergence of galago from its *Strepsirrhini* relative, mouse lemur. Tree begins with the *Euarchontoglines* common ancestor. In addition to mouse, rat, rabbit and tree shrew, we searched for but did not find *PGBD3* or MER85 in the assembled genomes of non-*Euarchontoglines* placental mammals (horse, cat, dog, cow, pig and sheep), nor in the more distantly related marsupial opossum and monotreme platypus. Arrowheads mark possible date of *PGBD3* invasions: As described in the text, the similarity of the galago consensus *PGBD3* sequence to human *PGBD3* suggests a contemporary origin for the elements, likely after the divergence of galago and mouse lemur (57 Mya) but before the divergence of marmoset and human (43 Mya). Approximate dates of divergence for primates are from Steiper et al. [78] and for non-primates from Springer et al. [79].

We were able to identify clear chimpanzee (*Pan troglodytes*) and Rhesus macaque (*Macaca mulatta*) homologs of *PGBD3* and all four of the pseudogenes by BLASTing the PGBD3 coding region against the recently-completed chimpanzee [40] and Rhesus [41] genomes. We also identified homologs of *CSB* and *PGBD3* in early assemblies of the orangutan (*Pongo abelli*) and white tufted-ear marmoset (*Callithrix jacchus*) genomes (Figure 6). All of these sequences predict that *PGBD3* will function as an alternative 3′ terminal exon to generate a CSB-PGBD3 fusion protein. Chimpanzee genomic sequences are approximately 98.8% identical to their human counterparts overall [40], and this was true for the four *PGBD3* pseudogenes and the 2 kb intronic regions immediately flanking the *PGBD3* coding region in *CSB* intron 5 (Table 1). As expected, the *CSB* protein coding region was more highly conserved between chimpanzee and human (99.5% DNA identity) than adjacent noncoding sequences. The *PGBD3* coding region was also much more highly conserved than noncoding sequence (99.7% DNA identity). For both genes, the degree of conservation lies outside the 95% confidence interval generated from the six noncoding regions we analyzed (98.4–99.3%, see Table 1). This was true for all four primate species examined — for example, *PGDB3* in marmoset, which last shared a common ancestor with humans approximately 43 Mya [36], is 96.1% identical in nucleotide sequence and 96.5% identical in amino acid sequence to its human homolog, compared to 95.2% and 94.1%, respectively, for *CSB* and 85.0–88.5% for noncoding nucleotide sequence (Table 1).

**Table 1:** Evolutionary Conservation of PGBD3.

Region	Human-chimpanzee % identity	Human-orangutan % identity^4^	Human-Rhesus % identity	Human-marmoset % identity
CSB coding cDNA^1^	99.5	98.5	97.2	95.2
PGBD3 coding cDNA	99.7	99.0	98.6	96.1
2 kb upstream of PGBD3 within CSB intron 5^2^	98.9	97.2	93.2	87.2
2 kb downstream of PGBD3 within CSB intron 5^2^	98.9	97.4	94.4	88.1
PGBD3P1	98.7	94.7	87.9	n/p
PGBD3P2	99.2	n/p	92.0	n/p
PGBD3P3(+)^3^	98.0	94.9	88.4	84.9
PGBD3P3(−)^3^	99.2	n/p	93.6	n/p
PGBD3P4	98.9	n/p	91.2	n/p
CSB protein	99.3	98.0	97.3	94.1
PGBD3 protein	99.7	99.3	98.8	96.5
Mean and (95% CI) of identity for six noncoding sequences	98.8 (98.4–99.3)	96.1 (94.5–97.6)	91.5 (88.9–94.2)	86.7 (85.0–88.5)

1 We compiled CSB coding cDNA sequence by mapping the human cDNA to primate genomic sequences.

2 2 kb of intron sequence was analyzed beginning immediately upstream and downstream of the inverted repeats flanking *PGBD3*.

3 The 3′ half of PGBD3P3 is inverted in human, chimp and Rhesus; conservation was calculated separately for the 5′ (+) and 3′ (−) portions of the pseudogenes.

4 Orangutan genome assembly includes sequencing gaps that obscure 380 nt of the latter portion of *CSB* exon 18 and 55 nt of *PGBD3*; these regions were omitted from identity calculations.

n/p, Not present in draft genome assembly.

A complementary method to estimate the degree to which a protein coding sequence is under purifying selection is to calculate the ratio of nonsynonymous (*Ka*, residue-altering) to synonymous (*Ks*, silent) nucleotide substitution rates; a low ratio suggests that the amino acid sequence is under strong purifying selection. We analyzed *CSB* and *PGBD3* coding sequences from human, chimp, orangutan, Rhesus and marmoset with the SNAP program [42], which implements the *Ka/Ks* algorithm of Nei et al. [43]. For comparison, the decayed *PGBD3* pseudogenes *PGBD3P1* and *PGBD3P3* have mean *Ka/Ks* values of 0.73 and 0.96, respectively, for pairwise comparisons between the various primate species (Table 2). *CSB*, presumably under purifying selection, has a mean *Ka/Ks* value of 0.21 (P<0.0001 *vs*. both *P1* and *P3*). The mean *Ka/Ks* for *PGBD3* is 0.12 (P<0.0001 *vs*. both *P1* and *P3*), consistent with the transposase being subject to purifying selection at least as strong as that for *CSB*. In fact, the mean *Ka/Ks* of *PGBD3* is significantly lower than that of *CSB* (P = 0.0006), though the difference between the entire fusion protein and *CSB* is not significant (P = 0.12).

**Table 2:** Ka/Ks Ratios for *CSB* and *PGBD3* between Primate Species.

	Human-chimp	Human-orangutan	Human-Rhesus	Human-marmoset	Mean of all ten* primate-primate comparisons (95% CI)
CSB	0.25	0.32	0.14	0.20	0.21 (0.18–0.25)
PGBD3	0.14	0.06	0.13	0.14	0.12 (0.10–0.14)
CSB-PGBD3 fusion	0.19	0.18	0.18	0.19	0.18 (0.17–0.19)
PGBD3P1	0.46	0.73	0.91	n/p	0.73 (0.63–0.83)
PGBD3P3	0.83	0.57	1.43	0.69	0.96 (0.73–1.2)

*: Six comparisons for PGBD3P1, which is not present in the marmoset genome draft.

We did not find a *CSB-PGBD3* homolog in the draft genome assemblies of two more distantly-related primates of the *Strepsirrhini* family: galago (*Otolemur garnetti*) and mouse lemur (*Microcebus murinus*), though the former may offer insights into the emergence of *PGBD3*. The mouse lemur genome contained no recognizable *PGBD3* or *MER85* elements. However, we found dozens of examples of each in galago although the two species diverged from a common ancestor only after the *Strepsirrhini* lineage separated from that of humans and marmosets (Figure 6) [44]. Despite this abundance, we confirmed by sequence alignment that the TTAA target site in galago *CSB* intron 5 is intact and empty. Moreover, of the eight galago *PGBD3*-like sequences we examined in detail, all are in an advanced state of decay, and all but one are more closely related to human *PGBD3* than to each other (Table S2). Interestingly, a consensus sequence of galago *PGBD3's* is as similar to human *PGBD3* (87.8% identity) as galago *CSB* exon sequences are to their human counterparts (87.6% identity), and both are significantly more identical than the individual *PGBD3*-like elements are to human *PGBD3* (see Table S2 for confidence intervals) - suggesting that the ancestor of these galago *PGBD3*-like sequences was closely related to conserved human *PGBD3*. The galago *PGBD3's* are equally similar to human *PGBD3* and this consensus (P = 0.61 by paired Student's T-test, see Table S2), consistent with divergence from a closely related ancestor. Together, these data suggest that an element closely related to the ancestral human *PGBD3* independently invaded the galago and human-marmoset lineages. Though invasion of the common galago-human ancestor by ancestral *PGBD3* would also explain the *PGBD3*-like sequences in galago, the monophyly of *Strepsirrhini* is well accepted [44] and it is unlikely that all traces of *PGBD3* and *MER85* would have been eradicated from the mouse lemur genome given their abundance in all genomes in which they are found. We conclude that an ancestral *PGBD3* element invaded *CSB* intron 5 at least 43 Mya, before human and marmoset diverged [36]; *PGBD3* was then conserved in the human-marmoset lineage because the CSB-PGBD3 fusion protein performs a selectable function (see Discussion) whereas the elements ultimately degenerated in galago where the random transpositions were either neutral or harmful.

### PiggyBac Survives as a Natural “Exon Trap”

The PiggyBac element has the hallmarks of a transposable element that has survived through evolution by functioning as a natural “exon trap”. In both cabbage looper moth and primates, the transposase ORF is flanked immediately upstream by a potential 3′ splice site (TTTTCTTGTTATAG in moth *PiggyBac*, CCTTTTTTCCGTTTTAG in *PGBD3*) and immediately downstream by a potential polyadenylation signal (AATAAATAAATAAA in moth *PiggyBac*, AATAAA in *PGBD3*). This 3′ splice site is perfectly conserved between human, chimpanzee, Rhesus, orangutan and marmoset (Figure S4), and in all five species *PGBD3* possesses a potential polyadenylation signal (Figure S5) despite evidence for strong selection against transcription of intragenic transposable elements [45]. Insertion of an element with these features into a host intron can generate an N-terminal fusion protein as observed for the *PGBD3* insertion into *CSB* intron 5 (Figure 1). Similarly, *PGBD1* and *PGBD2*, which are present in mouse and rat (though reduced to pseudogenes in mouse), also appear to have persisted as exon traps: The RefSeq human mRNAs include multiple upstream exons derived from the host gene, with the intact transposase encoded within a single large 3′ terminal exon. Indeed, the ability of the *T. ni* PiggyBac transposase to tolerate N-terminal fusions unlike the Sleeping Beauty, Tol2, and Mos1 transposases [15] is consistent with the genomic evidence that PiggyBac evolved as a 3′ terminal exon trap. Evolution as a 3′ exon trap may also explain the impressive host range of *T. ni* PiggyBac [46] because transcription of the element is driven by an efficient host promoter, instead of relying on fortuitous promoters or a universal species-independent promoter internal to the element itself.

### MER Elements and the Domestication of PGBD3

In contrast to *PGBD3*, the four *PGBD3*-related pseudogenes are all in an advanced state of decay (88–90% identity to *PGBD3*; see Figure S6). None of the pseudogenes contains an ORF longer than 62 codons and three exhibit major deletions or rearrangements. All are more closely related to *PGBD3* than to any of the other pseudogenes (Table S3), suggesting that all diverged from *PGBD3* itself or from a closely related common ancestor before the divergence of the human and Rhesus lineages. The left and right ends of *PGBD3* correspond to the left (100 nt) and right (40 nt) halves of the 140 nt MER85 repeat element [47], an arrangement also found in the four human *PGBD3* pseudogenes. We found 613 examples of MER85 elements in the human genome; in almost all cases, these were either intact left ends (403), intact right ends (119) or complete 140 nt elements (73). MER85 has been described as a nonautonomous transposable element derived from PiggyBac and presumably mobilized *in trans* by the PiggyBac transposase [1]; many other transposons have given rise to similar nonautonomous elements known collectively as “miniature inverted repeat transposable elements” or MITEs [12]. The similarly abundant MER75 and MER75B elements appear to be derived from *PGBD4*, although the PGBD4 transposase exon is no longer neatly flanked by its derivative elements as PGBD3 is by MER85. Consistent with previous estimates [48], neither MER75B nor MER85 has been significantly mobile since the divergence of human, chimpanzee and Rhesus. We found that 36 of 42 MER85 elements on human chromosome 1 had clear homologs on chromosome 1 of at least one of the other primates, as did 20 of 21 human MER75B elements. The few remaining unmatched human elements likely reflect incomplete sequences or recombination.

Most PiggyBac transposases have three conserved aspartic acid residues [13] which may be related to the metal-coordinating DDE motif found in the catalytic domain of many transposase and integrase families [49]. The most likely candidates for these conserved residues in PGBD3 [13] are identical in all five primates (human, chimp, orangutan, Rhesus and marmoset): D270, N352 and D467 (Figure S7). Strikingly, all four pseudogenes in human, chimp and Rhesus encode D at the second position (the draft orangutan and marmoset genomes do not yet include all *PGBD3* pseudogenes). Half of the galago *PGBD3*-like sequences we examined also encode D at this position, while the remainder harbor one of several changes (Figure S8). Together, this suggests that the feral ancestor of human *PGBD3* encoded a DDD motif, and that its domestication involved mutations that compromised mobility.

### Regulatory Patterns among MER85-Associated Genes

The exapted *mariner* transposase in the SETMAR fusion protein retains ancestral DNA binding activity despite attenuation or loss of transposase function [9]. We therefore asked whether genes located closest to MER85 elements might exhibit common themes or functions possibly reflecting a cis-regulatory function of the MER85 elements themselves or proteins that bind to them [50]. Using the ENSEMBL gene database, we located the transcription start site closest to each identified MER85 element (Table S4). The median distance between MER85 elements and transcription starts was 93 kb, similar to what is seen for other human repeats present in 500 to 4,000 copies [51]. Of the 613 MER85 elements, we selected the 585 that were less than 1 Mb from a transcription start site, well within the documented range of proximal enhancer elements [52]. We then used the L2L Microarray Analysis Tool [53] to search for expression patterns among these MER85-associated genes (Table S5). The strongest pattern to emerge was a striking similarity to genes down-regulated by UV irradiation in both normal and repair-deficient (XPB/CS, XPB/TTD) cells: Nine lists overlapped with P<0.02, and there was no similar finding among 1000 random-data simulations (Table S6). Intriguingly, the list of MER85-associated genes also overlapped significantly with the list of genes we had previously shown to be down-regulated by CSB (P = 0.012; corrected to P = 0.015 by random-data simulation) when hTERT-immortalized CSB-wt and CSB-null cell lines are compared [25]. There was no similar overlap with genes up-regulated by CSB. The most enriched Gene Ontology term was the Molecular Function “Glutamate Receptor Activity” (Table S7) reflecting association of MER85 with six glutamate receptors (GRM7, GRID1, GRID2, GRIK2, GRIN2A and GRIN2B) and two related GPCRs (7-fold enrichment, P = 1.6e-5; no similar finding among 1000 random-data simulations). Similar glutamate-related terms were the most enriched in the other Gene Ontology categories as well (data not shown).

## Discussion

We provide a combination of genomic, genetic, mRNA, and protein evidence that a CSB-PGBD3 fusion protein, generated by alternative splicing of *CSB* exon 5 to a *PGBD3* transposon within intron 5, is a major product of the *CSB/PGBD3* locus; that the fusion protein has been highly conserved in primates since the transposon was domesticated at least 43 Mya; that the fusion protein continues to be expressed in primary cells from three CS patients who lack functional CSB; and that nearly all CS-causing *CSB* mutations are located downstream of the exon 5/6 boundary in the ATPase and C-terminal domains of CSB protein, with the result that the fusion protein is predicted to be expressed in at least 21 of 24 characterized CS cell lines lacking functional CSB. The alternatively spliced CSB-PGBD3 mRNA was readily detectable by Q-RT-PCR, and was more abundant in all cell lines tested than full length CSB mRNA; the fusion mRNA had also been observed over a decade ago as an unexplained 3.4 kb polyadenylated RNA reacting with probes for the 5′ end but not the central region of *CSB* mRNA [54]. Consistent with our Q-RT-PCR data, we found by Western blotting that the CSB-PGBD3 fusion protein is abundant in a variety of primary and established CS and non-CS cells, and reacts as expected with both N-terminal CSB antibodies and a PGBD3-specific antibody.

### The CSB-PGBD3 Fusion Protein May Contribute to Cockayne Syndrome

**Figure 7:**
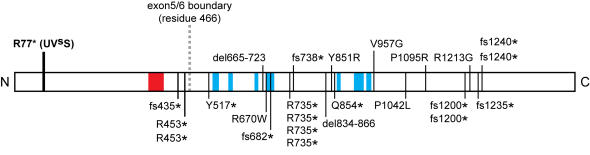
Schematic of CSB Mutations Associated with Human Disease. Of 24 patients with known *CSB* geneotypes and CS, COFS, or XP-DSC phenotypes, 21 have at least one allele that is predicted to express intact CSB-PGBD3 fusion protein. For each patient, the allele with the more C-terminal mutation is shown (e.g. patient CS1AN is a compound heterozygote for K337STOP and del834-866; only the latter is shown here). Some mutations are found in several patients. The location of the homozygous UV^s^S mutation that causes UV sensitivity but not CS [33] is shown for comparison. See Table S1 for a list of all patients and alleles. fs, frameshift; del, deletion; *, STOP. Red box is the CSB acidic domain; blue boxes are the ATPase subdomains.

Three mysteries have shaped thinking about Cockayne syndrome. First, the complete absence of CSB protein apparently does not cause CS, but rather a mild UV-sensitive syndrome with no developmental symptoms [33]. Yet all disease-associated *CSB* alleles identified to date are recessive; no dominant mutations are known. Second, nearly all *CSB* mutations that cause CS are located downstream of the exon 5/6 boundary (codon 466) in the ATPase and C-terminal regions of the 1493 residue protein (Figure 7; see Table S1 for details). And third, mouse models with either a truncating mutation similar to a severe human *CSB* allele (*CS1AN*; K337STOP) [55] or a *CSA* knockout [56] manifest the characteristic UV sensitivity of CS, as well as an unexpected susceptibility to skin cancer not observed for human *CSB* and *CSA* mutations, but only a subtle developmental phenotype. However, when the *CSB* defect is combined with an additional defect in an NER-GGR factor (*XPC* [57] or *XPA* [58]), mouse models do recapitulate the full CS-like phenotype including growth retardation, neurological dysfunction, and reduced life span.

The conserved CSB-PGBD3 fusion protein is expressed in both primary and established CS cells (Figures 2, 3, 5, and Figure S3), and could explain these mysteries if the fusion protein, which is advantageous in the presence of functional CSB (Tables 1 and 2), were detrimental in its absence. According to this hypothesis, mutations downstream of *CSB* exon 5 would cause CS by impairing expression of functional CSB without affecting expression of the fusion protein; nonsense and frameshift mutations upstream of exon 6 would not cause CS [33] because they would also abolish expression of the fusion protein; mutations that do cause CS would be recessive because functional CSB masks the effects of the CSB-PGBD3 fusion protein; and mouse models of severe *CSB* mutations or a *CSA* knockout would not exhibit the full range of CS symptoms because rodents lack the *PGBD3* insertion that generates the CSB-PGBD3 fusion protein.

Consistent with this hypothesis, 21 of the 24 molecularly characterized CS genotypes appear capable of expressing the CSB-PGBD3 fusion protein (Figure 7 and Table S1). We have also confirmed experimentally that the fusion protein continues to be expressed in primary cells from 3 severely affected CS patients (Figure 5) including patient CS1AN whose *CSB* genotype is known (Table S1). Only 3 of the 24 CS genotypes appear, on first sight, to be unable to express the fusion protein: the R453opal mutation found in first cousins CS1PV and CS3PV [59], and the +T1359 insertion mutation in patient CS10LO which causes a frameshift at residue 427 and termination at residue 435 [60]. However, all 3 of these CS genotypes could conceivably generate detectable levels of the CSB-PGBD3 fusion protein. UGA codons are often leaky [61] and can be suppressed by several natural tRNAs [62],[63]. Similarly, the existence and varying efficiency of programmed +1 and −1 frameshifting [64] suggests that frameshift mutations may sometimes be subject to a compensatory ribosomal frameshift that partially preserves the original reading frame. Indeed, ribosomal frameshifting is strongly dependent on context [65] which appears to be very “slippery” in the case of the +T1359 mutation (TTT TTC CCA to TTT TTT CCC) and could in principle increase the frequency of +1 frameshifts. Of course, leaky terminators and weak frameshifts might have been expected to rescue expression of both the CSB-PGBD3 fusion and full length CSB protein, but it should be kept in mind that the *CSB-PGBD3* and *CSB* mRNAs are alternatively spliced and polyadenylated transcripts with different intron/exon structures and different 3′ UTRs. The role of mRNA context and intron/exon structure in nonsense-mediated decay is still not fully resolved [66] and it is possible that the same mutation could differentially affect translation or degradation of the *CSB* and *CSB-PGBD3* mRNAs. Alternatively, the 3 anomalous patients (CS1PV, CS3PV, and CS10LO) may not express the fusion protein, but have other mutations or modifier genes which phenocopy the effect of the fusion protein.

If the CSB-PGBD3 fusion protein does indeed play a role in CS, the complex clinical presentation of the disease [26] might be explained by variable expression of the fusion protein in different individuals and cell types (Figure S3), or by the degree or nature of residual CSB activity. CS and genetically related syndromes like cerebro-oculo-facio-skeletal syndrome (COFS) and the DeSanctis-Cacchione variant of xeroderma pigmentosum (XP-DSC) could also be multifactorial, requiring two or more “hits” or perhaps modifier genes — consistent with mouse models showing that a *CSB* defect must be combined with a second defect in an NER-GGR factor (*XPC* [57] or *XPA* [58]) to generate a strong developmental phenotype.

A highly conserved and abundant protein which shares the first 5 exons of CSB is very likely to affect CSB-related cellular functions, but detailed functional characterization of the fusion protein will be required to understand how it could be detrimental in the absence of functional CSB protein. Unlike the ATPase domain of CSB encoded by sequences beyond the exon 5/6 boundary (Figure 1B) which is essential for DNA repair and chromatin remodeling, the N-terminal region encoded by CSB exons 1–5 is less well conserved and is apparently not essential either for transcription-coupled repair (TCR) or global genome repair (GGR) of UV-induced or bulky lesions [67]. Nonetheless, the possibility remains that in the absence of CSB, DNA repair complexes might recruit the CSB-PGBD3 fusion protein instead, blocking chromatin remodeling after attempted repair, preventing redundant repair pathways from accessing the damage, sequestering key repair factors, or even damaging the DNA if attempted repairs cannot be completed. This could also explain why CSA mutations are clinically indistinguishable from CSB mutations: Failure of CSA to target CSB [68] for ubiquitin-dependent degradation after CSB-dependent repair could have the same effect as the fusion protein in the absence of CSB — freezing repair complexes in place, and blocking subsequent events. Moreover, if the PGBD3 domain of the fusion protein targets CSB-dependent chromatin remodeling complexes to MER85 elements, loss of CSB might affect regulation of MER85-associated genes (Tables S4, S5, S6, S7) or enable MER85 elements themselves to sequester chromatin remodeling factors.

### Conservation of the Fusion Protein in Primate Lineages

The PGBD3 element in intron 5 of the *CSB* gene has not only been conserved for at least 43 Mya from marmoset to human, but the *PGBD3* element itself is at least as highly conserved as surrounding *CSB* sequences (Table 1). Moreover, synonymous changes are at least as abundant for *PGBD3* as for *CSB* in the human, chimp, orangutan, Rhesus and marmoset protein coding sequences (Table 2). We conclude that the initial *PGBD3* insertion was selected for a new function advantageous to the primate host, and the *CSB-PGBD3* fusion protein was thereafter subject to purifying selection to prevent loss of function.

The high correlation of homologous MER85 insertions in human, chimpanzee and Rhesus macaque on chromosome 1, and the absence of any lineage-specific *PGBD3* pseudogenes, suggests that neither *PGBD3* nor the related MER85 elements have been mobile since the three lineages diverged. These findings are consistent with several recent studies: an analysis of MER85 and MER75 sequence divergence by the Human Genome Sequencing Consortium [1], a comparative analysis of repetitive elements within the human, chimpanzee and Rhesus genomes [69], and an exhaustive study of DNA transposon activity in primates using ENCODE project genomic sequences [48]. The consistent D352 *versus* N352 difference in the putative catalytic DDD motif between decaying pseudogenes and *PGBD3* itself in all species (Figures S7 and S8) suggests that this change may have been critical for both the stability of *PGBD3* within *CSB* and for the demobilization of PGBD-related pseudogenes and MER elements derived from them. The same appears to be true for the domesticated *mariner* transposase of the SETMAR fusion protein where the catalytic DDD triad has mutated to DDN [9]. We speculate that both the *PGBD3* pseudogenes and the abundant MER85 elements are relics of a brief burst of activity when the *PGBD3* transposon, newly introduced into an ancestral primate genome, replicated without hindrance, and both spawned and propagated dependent MER elements.

Although complete and intact PGBD transposons are rare in all genomes examined [13], the abundance of MER elements suggests that infection of the primate lineage had the potential to get out of control. Indeed, the apparent independent infection of galago, whether by horizontal transfer from the contemporary human-marmoset ancestor or from an external source, and the dozens of degenerate *PGBD3*-like sequences generated by this infection, highlight the virulence of feral *PGBD3*. Insertional mutagenesis may have been the least of the dangers, as multiplying MER elements could have provided targets for genomic rearrangements mediated by the PGBD3 transposase — a well documented phenomenon for other DNA transposons with terminal inverted repeats such as *Drosophila* P-elements [70]. Domestication (i.e., insertion and fixation) of *PGBD3* within the *CSB* gene may have been the genetic response that restored genomic stability. Indeed, recruitment of the offending transposase itself in the form of a fusion protein has obvious advantages: The attenuated or inactivated transposase may simply occupy and occlude binding sites for the normal transposase — much as the absence of a germline-specific mRNA splice transforms the *Drosophila* P-element transposase into a somatic repressor of transposition [71] — or the fusion protein may actively guide host defense complexes to potential sites of excision, insertion, or rearrangement. It is also interesting to note that the *S. cerevisiae* homolog of *XPD*, known as *Rad3*, inhibits Ty1 retrotransposition [72]. CSB binds to several TFIIH subunits including XPD [17], suggesting a possible role for the N-terminal CSB domain of the CSB-PGBD3 fusion protein in silencing *PGBD3* family elements.

### Clues to a MER85 Gene Regulatory Network

Repression of PiggyBac and/or MER85 mobility may explain the initial domestication of *PGBD3* more than 43 Mya, but the CSB-PGBD3 fusion protein continues to be conserved and abundantly expressed in primates despite the passage of sufficient time to inactivate existing PGBD-related transposases. This suggests that the CSB-PGBD3 fusion protein may now be conserved for a new or secondary function. Noncoding elements account for the much of the genomic sequence under purifying selection in mammals [73], and many of these conserved noncoding sequences may be remnants of ancient transposons [50],[51]. The exaptation of SETMAR, fusing a SET histone methyltransferase domain to a *mariner*-like transposase, may have marked the emergence of a novel regulatory network based upon thousands of preexisting and now-selectable *mariner* elements [9]. Indeed, the exaptation of DNA-binding transposases has been proposed by Feschotte and Pritham [74] as “a pervasive pathway to create a genetic network [from] unlinked binding sites previously dispersed in the genome”. Our analysis of the genes closest to MER85 elements (Table S4) suggests that the CSB-PGBD3 fusion protein may have created just such a regulatory network based on MER85 elements. We had previously shown by expression microarray analysis that CSB protein has a general chromatin remodeling function which includes the maintenance of transcriptional silencing; specifically, loss of CSB phenocopied conditions that disrupt chromatin structure such as treatment with inhibitors of histone deacetylation and DNA methylation, and defects in poly(ADP-ribose)-polymerase [25]. Surprisingly, many of the CSB-repressed genes are associated with MER85 elements (Table S5, “*csb_reliable_up*” database list). Just as striking was the association of MER85 elements with genes that are repressed following UV irradiation (Tables S5 and S6); UV is known to cause nuclear translocation of CSA [75] which may in turn be required for full CSB function. Thus, recruitment of CSB or CSB-associated factors to MER85 elements by the CSB-PGBD3 fusion protein, perhaps in combination with independently transcribed PGBD3 transposase (Figures 1 and 4), may not only inhibit PGBD-mediated transposition, but also transcription of neighboring genes. The overabundance of neuronal genes — specifically glutamate receptors — among those closest to MER85 elements (Table S7) is particularly intriguing because CS exhibits a strong neurodegenerative component. Sarkar et al. [13] note that the independent domestication of PiggyBac in nearly all metazoan lineages suggests that these transposable elements “have repeatedly been turned to advantage by the host.” We suggest that this is a natural consequence of the PiggyBac lifestyle as a 3′ terminal exon trap in which the transposase ORF is flanked by 3′ splice site and polyadenylation signals (Figure 1 and Figures S4 and S5), and the activity of the transposase protein readily tolerates N-terminal fusions [15]. We do not yet know why the CSB-PGBD3 fusion protein has been selected and maintained in the primate lineage for over 43 My, but the answers will undoubtedly shed light on both CSB function and the longevity of PiggyBac transposases from cabbage looper moths to humans [13].

## Materials and Methods

### Cell Lines and Culture Conditions

HT1080 (human fibrosarcoma), MRC5 (human embryonic lung fibroblast) and Saos-2 (human osteosarcoma) cell lines, along with primary CS cells GM0010903 and GM0010905 were obtained from repositories. WI38 human embryonic lung fibroblasts were immortalized by PG-13/neo retroviral transduction of hTERT cDNA [76]. Immortalized CSB (CS1AN) fibroblasts expressing either wild-type *CSB* cDNA (CSB-wt line) or enhanced green fluorescent protein (CSB-null line) were generated as described [25]. HeLa, WI38/hTERT, and CS1AN-derived lines were cultured in MEMα media with 10% fetal bovine serum plus supplements (Gibco). Selection for expression of hTERT, CSB, and enhanced green fluorescent protein was maintained with 1 mg/ml G418 and 0.5 µg/ml puromycin, respectively. Cells were passaged by a wash in Puck's EDTA followed by trypsinization. HT1080 cells were cultured in MEMα media with 10% fetal bovine serum, and passaged by a wash in PBS followed by trypsinization.

### Real-Time Quantitative RT-PCR

Total RNA was harvested directly from adherent cells with Trizol reagent (Ambion). Synthesis of cDNA was primed with oligo(dT) and carried out using Superscript II reverse transcriptase (Invitrogen). Each real-time reaction consisted of cDNA template from 20–50 ng of total RNA, 300 nM 5′ and 3′ gene-specific primers, and 1× SYBR Green master mix (Applied Biosystems) in 20 µl total reaction volume. All reactions were performed in triplicate using the DNA Engine Opticon real-time PCR system (MJ Research). Relative differential expression was calculated from mean threshold cycle difference among the three replicate reactions. Products were visualized by pooling the three replicate reactions, purifying and concentrating over a QIAquick column (Qiagen), and running half of the total sample on a 1.0% agarose gel stained with ethidium bromide. Primer sequences are available on request.

### Sequence Comparison of PiggyBac Genes

Pairwise alignments and comparisons of analogous sequences were performed by Needleman-Wunsch global alignment, as implemented in EMBOSS *needle*. Overhanging ends were excluded from the identity calculations. We compared only homologous sequence regions: For example, we ignored the truncations of several *PGBD3* pseudogenes when calculating their homology to *PGBD3*. Coding region identity was calculated from translation start to stop codons. Pseudogene identities were calculated from the 3′ SS (or start of homology) to the stop codon (or end of homology). We used RepBase *RepeatMasker* to identify the flanking MER85 and MER75B elements of *PGBD3* and *PGBD4*, respectively. To determine if the conservation of the *PGBD3* and *CSB* coding regions is statistically significant, we analyzed the conservation of six noncoding sequences for comparison: 2 kb of intron sequence beginning both immediately upstream and downstream of the inverted repeats flanking *PGBD3*, and the four *PGBD3* pseudogenes. We determined the conservation of each of these six sequences individually by pairwise alignment between species using *needle*. We calculated a mean identity of all six and then used the inverted t-distribution to generate a confidence interval. The conservation of the *PGBD3* and *CSB* coding regions was considered significant if the identity fell outside the 95% confidence interval of conservation for these six noncoding regions; this calculation is not dependent on the length of the query sequences. In order to determine whether MER85 and MER75B elements have been mobile since the divergence of the three primates, we used NCBI *megaBLAST* to identify all MER85 and MER75B elements on human, chimpanzee and Rhesus chromosome 1 (June 2006 NCBI sequence releases), based on the consensus sequence for these elements in RepBase Update [47]. We then extracted 1 kb of the surrounding sequence for each element, and used EMBOSS *needle* to align every such human sequence pairwise with every sequence from chimpanzee and monkey. Marmoset (version 2.0.2, released June 2007) and orangutan (version 2.0.2, released July 2007) preliminary genome assemblies were downloaded from the Washington University Genome Sequencing Center. Mouse lemur (draft v2, released June 2007), galago (draft v1, released June 2006) and tree shrew (draft v1, released June 2006) genome sequences were downloaded from the Broad Institute Mammalian Genome Project. *Ka/Ks* analysis was performed using SNAP (Synonymous Nonsynonymous Analysis Program) from the HIV Database at Los Alamos National Laboratories (USA) [42]. The significance of differences in *Ka/Ks* values was calculated with the Student's T-test using a two-tailed distribution and an assumption of unequal variance. All sequences and alignments used in this study are available on request.

### Analysis of MER-Associated Genes

MER85 elements were identified in the March 2006 release of the NCBI human genome sequence by using NCBI *megaBLAST* to query each complete chromosome sequence for the RepBase MER85 consensus sequence. The start site of each element was matched to the closest start site of an HGNC-named gene from the ENSEMBL database. The resulting list of genes, excluding those located >1 Mb from their associated MER85 element, was analyzed with the 2007.1 release of the L2L Microarray Analysis Tool, including several unreleased lists representing CSB-regulated genes. The list of all HGNC-named genes in the ENSEMBL database was used as the null set. The P values generated by L2L were validated using random-data simulations as described previously [25]. Briefly, we randomly selected 1000 lists of genes from the null set, each the same size as the list of MER85-associated genes, and ran each through an identical L2L analysis. These random-data results were mined for the frequency of the outcomes seen in the analysis of MER85-associated genes.

### Western Blots

GM00739B/hTERT cells were transfected in 100 mm tissue culture plates with 10 µg of plasmid constructs using 15 µl of Fugene 6 reagent (Roche). After 48 h, cells were washed with PBS and harvested by scraping. Cell pellets were resuspended in 100 µl of SDS loading buffer (25 mM Tris, pH 6.8, 2% SDS, 0.1% bromephenol blue, 10% sucrose, 0.12 M β-mercaptoethanol), sonicated to shear DNA, and denatured by heating at 95°C for 10 min. Non-transfected plates of HT1080 and WI-38/hTERT cells were harvested in the same manner. Proteins were separated on a 6% gel by SDS-PAGE using the Mini-Protean 3 Cell (BioRad) in a Tris/glycine/SDS buffer (1.5 g/l Tris base, 7.2 g/l glycine, 1% SDS). Proteins were transferred to a PDVF membrane in 25 mM Tris, 192 mM glycine, and 20% methanol buffer using a Mini Trans-Blot Cell (BioRad). After transfer, the PVDF membranes were blocked for 2 h at room temperature in TBST (50 mM Tris, pH 7.4, 150 mM NaCl, 0.05% Tween 20) plus 5% nonfat dry milk. The membrane was then incubated at room temperature in TBST plus 5% nonfat dry milk for 2 h with a 1∶1000 dilution of primary antibody, washed twice for 10 min each, incubated for 1 h with a 1∶5000 dilution of HRP-conjugated secondary antibody (Santa Cruz Biotechnology), and finally washed 4 times for 10 min each in TBST alone. Chemiluminescent detection was performed using the ECL Plus™ Western Blotting Detection System (Amersham) and Kodak X-Omat Blue film. Anti-CSB antibodies were generated in our laboratory as rabbit polyclonals raised to the C-terminal 158 amino acids or N-terminal 240 amino acids of CSB expressed as bacterial GST fusion proteins. Anti-GST antibodies were removed from the serum by passage over a GST column. Anti-PGBD3 antibody was purchased from AVIVA Systems Biology, catalog number ARP36534.

### GenBank Accessions for Primate Sequences

Human PGBD3 and the four PGBD3 pseudogenes are present in the NCBI Entrez Gene database, but have not yet been curated in the chimpanzee or Rhesus genomes. The accessions and approximate indicies for the coding region sequences used in this study are as follows:

1. Chimpanzee
2. PGBD3: NW_112875 REGION: 1929225..1927305 (chr 10)
3. PGBD3P1: NW_114825 REGION: 5794955..5793042 (chr 12)
4. PGBD3P2: NW_107030 REGION: 2131567..2132809 (chr 5)
5. PGBD3P3: NW_114852 REGION: 191259..192848 (chr 12)
6. PGBD3P4: NW_105918 REGION: 8439037..8440924 (chr 4)
7. Rhesus
8. PGBD3: NW_001124201 REGION: 674139..676053 (chr 9)
9. PGBD3P1: NW_001096629 REGION: 99414..98334 (chr 11)
10. PGBD3P2: NW_001120954 REGION: 9223297..9222041 (chr 6)
11. PGBD3P3: NW_001096663 REGION: 36134..37168 (chr 11)
12. PGBD3P4: NW_001118143 REGION: 11277269..11279192 (chr 5)

## Supporting Information

Table S1 CSB mutations associated with human disease.
(0.06 MB DOC)

Table S2 Comparison of galago PGBD3-like sequences with human PGBD3.
(0.06 MB DOC)

Table S3 Sequence identity between PGBD3 and pseudogenes.
(0.04 MB DOC)

Table S4 Locations of human MER85 elements and MER85-associated genes.
(0.09 MB XLS)

Table S5 L2L results comparing MER85-associated genes to the L2L microarray database, with P values corrected by random-data simulation.
(0.17 MB XLS)

Table S6 L2L microarray database results analyzed by groups of similar database lists.
(0.02 MB XLS)

Table S7 L2L results comparing MER85-associated genes to Gene Ontology Molecular Function terms, with P values corrected by random-data simulation.
(0.17 MB XLS)

Figure S1 Quantitation of the relative abundance of CSB and CSB-PGBD3 fusion transcripts in HeLa cDNA. (A) Ratio of abundance between similarly sized CSB and fusion PCR products, assayed by real time RT-PCR. The average ratio of all eighteen fusion:CSB comparisons is 2.0:1. (B) Expected sizes of all PCR products.
(1.07 MB TIF)

Figure S2 Fusion mRNA is more abundant than CSB in other cell lines. Three pairs of primer combinations (A1–D1, A2–D2 and B1–E1) were tested on cDNA from CSB-null, CSB-wt and WI38/hTERT cell lines. In all cases, the fusion PCR products were substantially more abundant than the corresponding CSB products as quantified by real time RT-PCR.
(0.65 MB TIF)

Figure S3 Additional Western blots for CSB and the fusion protein. (A) Western blot using a Cterminal antibody against CSB reveals only the expected major band for full length CSB(filled arrowhead) in MRC5 (SV40-immortalized human fetal lung fibroblast), E61ANd(GM00739B fibroblasts from compound heterozygote CS1AN, immortalized by SV40 and rescued by wt CSB cDNA [1]), Saos-2 (human osteosarcoma) and HT1080 (human fibrosarcoma); full length CSB is not seen in CS1AN/SV (the SV40-immortalized but unrescued parent of E61ANd). The >250 kDa species (labeled X) is seen only with Cterminal antibody and correlates with the abundance of full length CSB, but is also weakly expressed in the CS1AN/SV line lacking full length CSB; X may be a modified form of full length CSB, and weak expression in CS1AN/SV may indicate that one or both of the nonsense mutations in this compound heterozygote are leaky [2]. (B) Western blot using an N-terminal antibody against CSB reveals both full length CSB (filled arrowhead) and a second major band corresponding to the fusion protein (hollow arrowhead) in MRC5, Saos-2, and HT1080. We were also able to detect the fusion protein, but not full-length CSB, in SV40-immortalized primary fibroblasts from CSB patient CS1BE (derived from GM01629; data not shown). In agreement with our data, a band corresponding to the CSB-PGBD3 fusion protein was previously seen in the normal SV40-transformed WI38VA13 line [3] and in normal hTERT-immortalized BJ1 fibroblasts [4]. Although present in our CS1AN/hTERT line (Fig. 3), the fusion protein appears to be absent in the CS1AN/SV line (see also [3],[4]) and its derivatives, suggesting that SV40 immortalization may suppress the fusion protein. (C) Western blots using an N-terminal CSB antibody reveal only the fusion protein (hollow arrowhead) and not fulllength CSB (filled arrowhead) in three different primary CSB cells. GM00739B is derived from patient CS1AN and is the parent of our hTERT-immortalized derivatives (CSB-wt and CSB-null lines), thus demonstrating that the fusion protein is not an artifact of immortalization. GM10903 and GM10905 are from two patients diagnosed with the DeSanctis-Cacchione variant of XP (XP/DCS; XP61SF and XP63SF in Table S3). The same homozygous R735STOP mutation shared by both patients is also associated with classical CS in another patient (CS1TAN) and the clinical overlap between CSB-XP/DCS and CSB-CS is substantial, including photosensitivity (but not skin cancers), mental retardation and severe growth failure [5]. The fusion protein might therefore contribute to the entire spectrum of CSB-associated disease. Markers are in kDa. The apparent sizes of full-length CSB and the fusion protein in these panels and in Figures 3 and 4 are similar but not identical because the gels were run under various conditions and using different markers. Figures 3 and 4 should be considered the size standard; these supporting panels assess the relative abundance of CSB and the fusion protein in different cells and cell lines. 1. Troelstra C, van Gool A, de Wit J, Vermeulen W, Bootsma D, et al. (1992) ERCC6, a member of a subfamily of putative helicases, is involved in Cockayne's syndrome and preferential repair of active genes. Cell 71: 939–953. 2. Keeling KM, Lanier J, Du M, Salas-Marco J, Gao L, et al. (2004) Leaky termination at premature stop codons antagonizes nonsense-mediated mRNA decay in S. cerevisiae. Rna 10: 691–703. 3. Horibata K, Iwamoto Y, Kuraoka I, Jaspers NG, Kurimasa A, et al. (2004) Complete absence of Cockayne syndrome group B gene product gives rise to UV-sensitive syndrome but not Cockayne syndrome. Proc Natl Acad Sci U S A 101: 15410–15415. 4. Groisman R, Kuraoka I, Chevallier O, Gaye N, Magnaldo T, et al. (2006) CSAdependent degradation of CSB by the ubiquitin-proteasome pathway establishes a link between complementation factors of the Cockayne syndrome. Genes Dev 20: 1429–1434. 5. Greenhaw GA, Hebert A, Duke-Woodside ME, Butler IJ, Hecht JT, et al. (1992)Xeroderma pigmentosum and Cockayne syndrome: overlapping clinical and biochemical phenotypes. Am J Hum Genet 50: 677–689.
(4.58 MB TIF)

Figure S4 Conservation of the 3′ splice site in PGBD3. (A) Conserved 3′ splice site in the genomic sequence of PGBD3 and pseudogenes for human (Hs, Homo sapiens), chimpanzee (Pt, Pan troglodytes), Rhesus (Mm, Macaca mulatta), marmoset (Ct, Callithrix jacchus) and orangutan (Pa, Pongo abelli), organized by gene. Uppercase letters represent exon sequence; ATG at the 5′ end is the start codon of the PiggyBac ORF. Notably, the entire sequence shown is perfectly conserved in PGBD3 from all three species. (B) As for (A), organized by species to show the variations between PGBD3 and its pseudogenes.
(1.47 MB TIF)

Figure S5 Conservation of the polyadenylation signal in PGBD3. (A) Conserved polyadenylation signals in the genomic sequence of PGBD3 and pseudogenes for human (Hs), chimpanzee (Pt), Rhesus (Mm), marmoset (Ct) and orangutan (Pa), organized by gene. Uppercase letters represent the AAUAAA motif. Dashes indicate pseudogenes for which the 3′ end (including the polyadenylation site) is no longer present. (B) As for (A), organized by species to show the variations between PGBD3 and its pseudogenes.
(1.17 MB TIF)

Figure S6 Schematic of PGBD3 element and the relationship with PGBD3 pseudogenes. The 5′ and 3′ ends of PGBD3 correspond to the 5′ arm (100 nt) and 3′ arm (40 nt) of a MER85 element (140 nt). The 13 nt inverted repeats of MER85 define the boundaries of the element, which is flanked by a TTAA target site duplication. Only two pseudogenes span the entire element, but all pseudogenes with intact ends share the same flanking features as PGBD3, and exhibit no homology to PGBD3 beyond the TTAA duplications. The 3′ end of PGBD3P3 is inverted, but the inverted sequence is as similar to PGBD3 as the remainder. An AluSx SINE has inserted into PGBD3P4 between the MER85 element and the 3′ SS. Also note two fragments (yellow) that appear to be derivatives of the left end of MER85, spanning 14 and 25 nt. The fragments each include the 5 innermost nt of the MER85 inverted repeat, but also share 8 further nt of unique flanking inverted repeat. Chimpanzee and Rhesus PGBD3 are structurally identical to human PGBD3, including all of the elements displayed. The chimpanzee pseudogenes are essentially identical to their human homologs. The Rhesus pseudogenes are more highly degraded, particularly at the 3′ end.
(0.61 MB TIF)

Figure S7 The putative catalytic motif is DDD in all pseudogenes, but DND in all PGBD3s. PiggyBac transposases share a DDD motif [1] that may be analogous to the metalcoordinating DDE motif common to other transposase families [2]. The most likely candidates for this motif in human PGBD3 are D270, N352 and D467. N352 is the result of a G to A mutation that occurred after generation of the four pseudogenes; all pseudogenes encode D at this position. (A) Genomic sequence of the three putative catalytic residues of PGBD3 and pseudogenes for human (Hs), chimpanzee (Pt), Rhesus (Mm), marmoset (Ct) and orangutan (Pa) organized by gene. Uppercase letters are the codons for the three residues. Dashes indicate pseudogenes for which the homologous sequence is no longer present; in addition, a 49 nt gap in the genome sequence of orangutan obscures D270. (B) As for (A), organized by species to show the variations between PGBD3 and its pseudogenes.
(1.30 MB TIF)

Figure S8 Consensus sequence of putative catalytic motif is DDD in galago PGBD3-related sequences. Six of seven sequences encode D at position 270, and six of seven encode D at 467. At position 352, four encode D while three encode K, G or N. Numbers designtating the PGBD3-like sequences represent the galago (Otolemur garnetti, Og) genomic contig on which the sequence is located.
(0.36 MB TIF)
