# Supplementary material for: An Abundant Evolutionarily Conserved CSB-PiggyBac Fusion Protein Expressed in Cockayne Syndrome
Source: PLoS Genet. 2008 Mar 21;4(3):e1000031. doi: 10.1371/journal.pgen.1000031 (PMC2268245; doi:10.1371/journal.pgen.1000031)
Supplement: Table S1 — CSB mutations associated with human disease. (0.06 MB DOC) [file pgen.1000031.s001.doc]

**Table S1**: CSB mutations associated with human disease [1-6]

| Patient | Allele 1 | Allele 2 | Reference | Notes |
| --- | --- | --- | --- | --- |
| 25627 | R735* | R453* | Mallery, 1998 |  |
| COFS02MA | fs1240* | fs1240* | Meira, 2000 | COFS |
| COFS03MA | fs1240* | fs1240* | Meira, 2000 | COFS |
| CS10BR | fs1235* | fs1235* | Mallery, 1998 |  |
| **CS10LO** | **fs435*** | **fs435*** | **Mallery, 1998** |  |
| CS1ABR | fs738* | fs738* | Mallery, 1998 |  |
| CS1AN | del834-866 | K337* | Troelstra, 1992 |  |
| CS1BE | R670W | fs1200* | Mallery, 1998 |  |
| CS1BO | fs541* | P1095R | Mallery, 1998 |  |
| CS1IAF | V957G | V957G | Mallery, 1998 |  |
| CS1MA | del665-723 | del665-723 | Mallery, 1998 |  |
| **CS1PV** | **R453*** | **R453*** | Colella, 1999 |  |
| CS1TAN | R735* | R735* | Mallery, 1998 |  |
| CS2BE | del665-723 | fs1200* | Mallery, 1998 |  |
| CS2BI | P1042L | R670W | Mallery, 1998 |  |
| CS2PV | fs368* | fs682* | Colella, 1999 |  |
| CS2TAN | Y517* | Y517* | Mallery, 1998 |  |
| **CS3PV** | **R453*** | **R453*** | **Colella, 1999** |  |
| CS3TAN | Y851R | Y851R | Mallery, 1998 |  |
| CS4BR | Q184* | R670W | Mallery, 1998 |  |
| CS7TAN | R1213G | R1213G | Mallery, 1998 |  |
| CS8BR | Q854* | Q854* | Mallery, 1998 |  |
| **UVS1KO** | **R77*** | **R77*** | **Horibata, 2004** | **UVsS** |
| XP61SF | R735* | R735* | Colella, 2000 | XP-DSC |
| XP63SF | R735* | R735* | Colella, 2000 | XP-DSC |

## Patients reported to have two alleles with mutations upstream of exon 5/6 boundary are in bold; see text for discussion. Additional polymorphisms that are unlikely to contribute to the CS phenotype have been reported in several patients [1]. fs, frameshift; del, deletion; *, STOP; COFS, Cerebro-oculo-facio-skeletal syndrome; UVsS, UV-sensitive syndrome; XP-DSC, DeSanctis-Cacchione variant of xeroderma pigmentosum.

1. Mallery DL, Tanganelli B, Colella S, Steingrimsdottir H, van Gool AJ, et al. (1998) Molecular analysis of mutations in the CSB (ERCC6) gene in patients with Cockayne syndrome. Am J Hum Genet 62: 77-85.

2. Troelstra C, van Gool A, de Wit J, Vermeulen W, Bootsma D, et al. (1992) ERCC6, a member of a subfamily of putative helicases, is involved in Cockayne's syndrome and preferential repair of active genes. Cell 71: 939-953.

3. Horibata K, Iwamoto Y, Kuraoka I, Jaspers NG, Kurimasa A, et al. (2004) Complete absence of Cockayne syndrome group B gene product gives rise to UV-sensitive syndrome but not Cockayne syndrome. Proc Natl Acad Sci U S A 101: 15410-15415.

4. Colella S, Nardo T, Mallery D, Borrone C, Ricci R, et al. (1999) Alterations in the CSB gene in three Italian patients with the severe form of Cockayne syndrome (CS) but without clinical photosensitivity. Hum Mol Genet 8: 935-941.

5. Colella S, Nardo T, Botta E, Lehmann AR, Stefanini M (2000) Identical mutations in the CSB gene associated with either Cockayne syndrome or the DeSanctis-cacchione variant of xeroderma pigmentosum. Hum Mol Genet 9: 1171-1175.

6. Meira LB, Graham JM, Jr., Greenberg CR, Busch DB, Doughty AT, et al. (2000) Manitoba aboriginal kindred with original cerebro-oculo- facio-skeletal syndrome has a mutation in the Cockayne syndrome group B (CSB) gene. Am J Hum Genet 66: 1221-1228.
