# Supplementary material for: An Abundant Evolutionarily Conserved CSB-PiggyBac Fusion Protein Expressed in Cockayne Syndrome
Source: PLoS Genet. 2008 Mar 21;4(3):e1000031. doi: 10.1371/journal.pgen.1000031 (PMC2268245; doi:10.1371/journal.pgen.1000031)
Supplement: Table S2 — Comparison of galago PGBD3-like sequences with human PGBD3. (0.06 MB DOC) [file pgen.1000031.s002.doc]

**Table S2:** Comparison of galago *PGBD3*-like sequences with human *PGBD3*.

| Galago source contig | % identity to human *PGBD3* | % identity to galago consensus | | % identity to 112983 | % identity to 112895 | % identity to 81560 | % identity to 116009 | % identity to 13526 | % identity to 93566 | % identity to 99260 | % identity to 7934 |
| --- | --- | --- | --- | --- | --- | --- | --- | --- | --- | --- | --- |
| 112983 | 87.7 | 80.8 | | n/a | 78.7 | 80.9 | 78.8 | 76.8 | 80.2 | 71.2 | 67.2 |
| 112895 | 82.2 | 86.0 | | 78.7 | n/a | 75.1 | 76.6 | 74.3 | 72.1 | 67.2 | 66.8 |
| 81560 | 82.1 | 81.0 | | 80.9 | 75.1 | n/a | 75.5 | 74.6 | 74.3 | 68.9 | 65.2 |
| 116009 | 81.8 | 81.9 | | 78.8 | 76.6 | 75.5 | n/a | 76.0 | 72.7 | 70.3 | 68.3 |
| 13526 | 79.7 | 79.9 | | 76.8 | 74.3 | 74.6 | 76.0 | n/a | 72.1 | 68.0 | 67.6 |
| 93566 | 79.1 | 77.9 | | 80.2 | 72.1 | 74.3 | 72.7 | 72.1 | n/a | 67.4 | 64.6 |
| 99260 | 74.4 | 73.3 | | 71.2 | 67.2 | 68.9 | 70.3 | 68.0 | 67.4 | n/a | 60.6 |
| 7934 | 70.2 | 71.8 | | 67.2 | 66.8 | 65.2 | 68.3 | 67.6 | 64.6 | 60.6 | n/a |
|  | P = 0.61* | | |  |  |  |  |  |  |  |  |
| Mean (95% CI) | 79.6  (76.0-83.3) | | 79.1  (75.9-82.3) |  |  |  |  |  |  |  |  |

* P value calculated by paired, two-tailed Student’s T-test.

Note: Galago *PGBD3* consensus sequence is 87.8% identical to human *PGBD3*. We identified the complete sequences of *CSB* exons 5-7 and 14-18 in the draft galago genome assembly; these were 87.1% and 88.0% identical, respectively, and 87.6% identical in aggregate, to the homologous regions of the human *CSB* cDNA.
