# Supplementary material for: An Abundant Evolutionarily Conserved CSB-PiggyBac Fusion Protein Expressed in Cockayne Syndrome
Source: PLoS Genet. 2008 Mar 21;4(3):e1000031. doi: 10.1371/journal.pgen.1000031 (PMC2268245; doi:10.1371/journal.pgen.1000031)
Supplement: Table S3 — Sequence identity between PGBD3 and pseudogenes. (0.04 MB DOC) [file pgen.1000031.s003.doc]

**Table S3**: Sequence identity between PGBD3 and pseudogenes

|  | PGBD3 | PGBD3P1 | PGBD3P2 | PGBD3P3(+)* | PGBD3P3(-)* | PGBD3P4 |
| --- | --- | --- | --- | --- | --- | --- |
| PGBD3 | - | 90.5 | 89 | 90.4 | 89.6 | 88.2 |
| PGBD3P1 | 90.5 | - | 85.1 | 87.4 | 83.4 | 85.5 |
| PGBD3P2 | 89 | 85.1 | - | 85.1 | 86.5 | 83.7 |
| PGBD3P3(+)* | 90.4 | 87.4 | 85.1 | - | N/A | 84.3 |
| PGBD3P3(-)* | 89.6 | 83.4 | 86.5 | N/A | - | 83.3 |
| PGBD3P4 | 88.2 | 85.5 | 83.7 | 84.3 | 83.3 | - |

* The 3’ half of PGBD3P3 is inverted in all three species; conservation was calculated separately for the 5’ (+) and 3’ (-) portions of the pseudogenes.

N/A, not applicable.
