# Supplementary figures and images for: An Abundant Evolutionarily Conserved CSB-PiggyBac Fusion Protein Expressed in Cockayne Syndrome
Source: PLoS Genet. 2008 Mar 21;4(3):e1000031. doi: 10.1371/journal.pgen.1000031 (PMC2268245; doi:10.1371/journal.pgen.1000031)

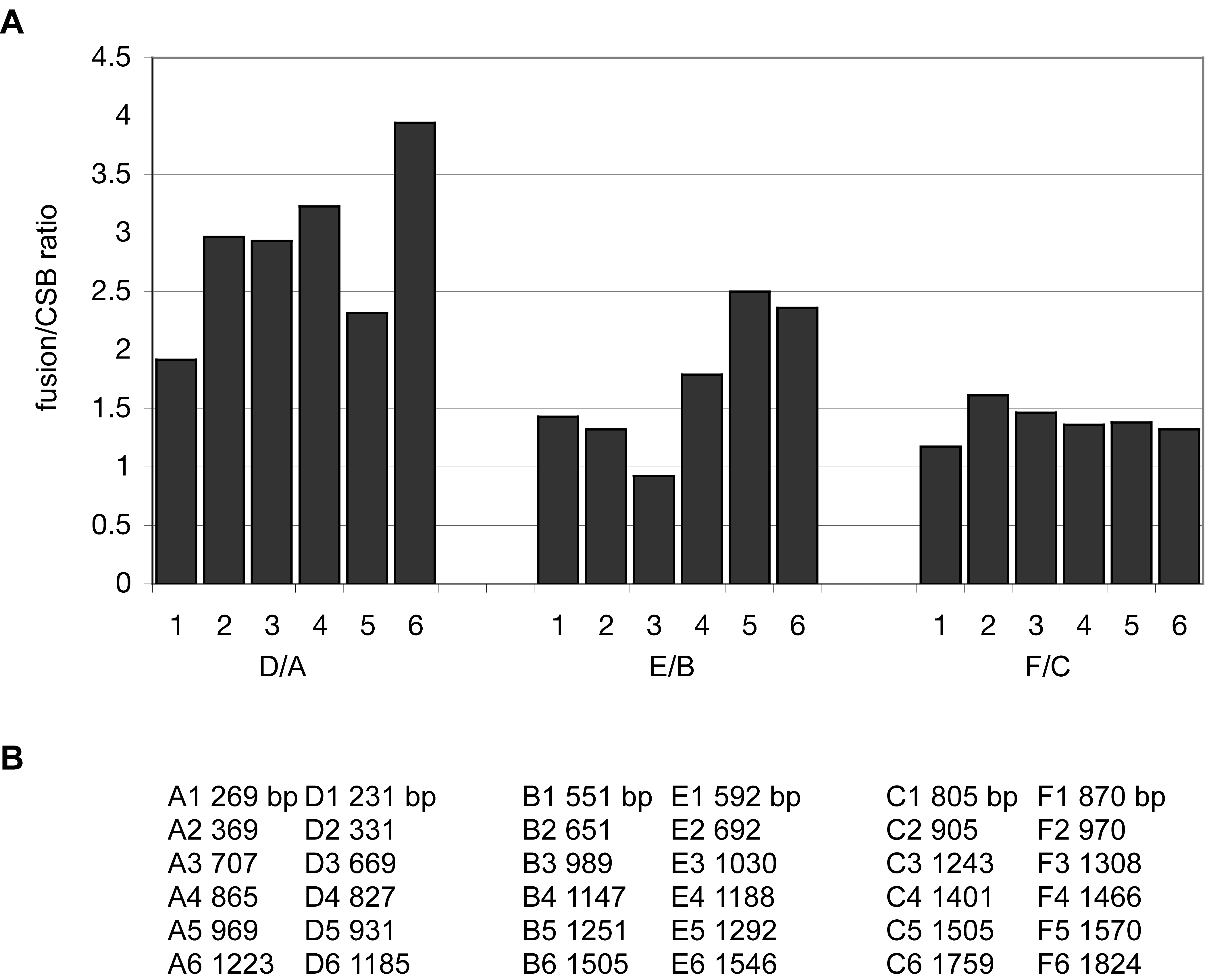

Supplement: Figure S1 — Quantitation of the relative abundance of CSB and CSB-PGBD3 fusion transcripts in HeLa cDNA. (A) Ratio of abundance between similarly sized CSB and fusion PCR products, assayed by real time RT-PCR. The average ratio of all eighteen fusion:CSB comparisons is 2.0:1. (B) Expected sizes of all PCR products. (1.07 MB TIF) [file pgen.1000031.s008.tif]

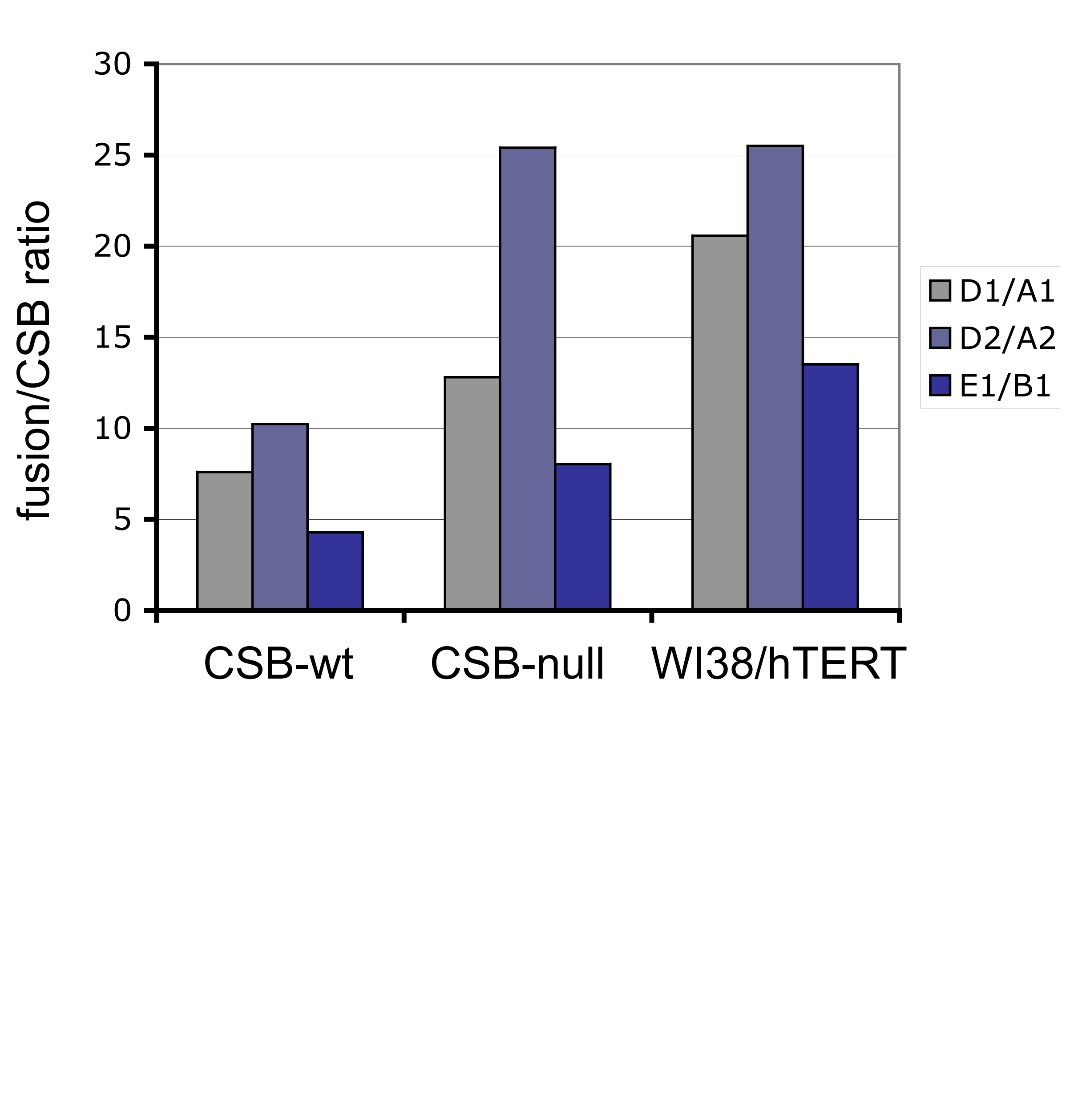

Supplement: Figure S2 — Fusion mRNA is more abundant than CSB in other cell lines. Three pairs of primer combinations (A1–D1, A2–D2 and B1–E1) were tested on cDNA from CSB-null, CSB-wt and WI38/hTERT cell lines. In all cases, the fusion PCR products were substantially more abundant than the corresponding CSB products as quantified by real time RT-PCR. (0.65 MB TIF) [file pgen.1000031.s009.tif]

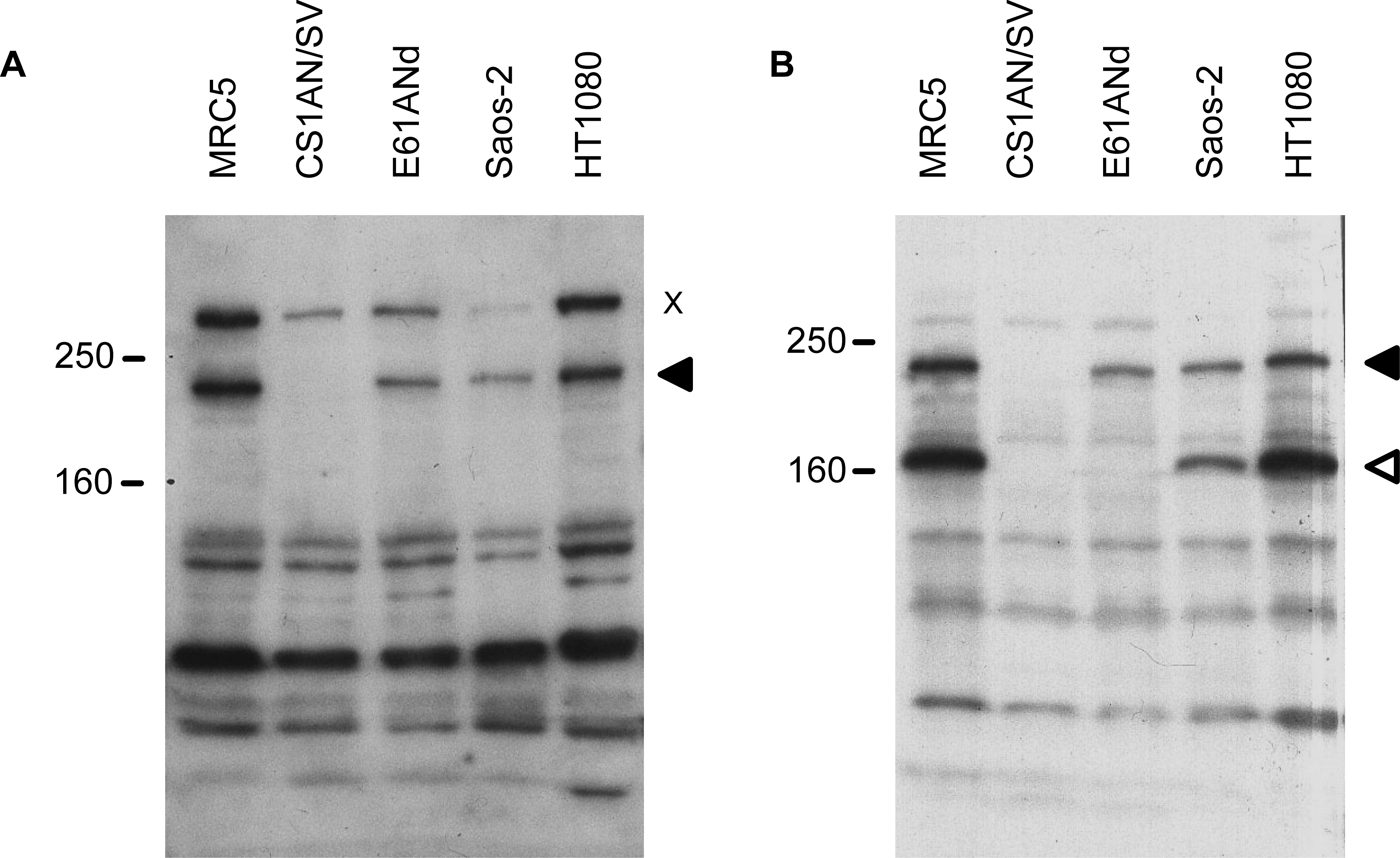

Supplement: Figure S3 — Additional Western blots for CSB and the fusion protein. (A) Western blot using a Cterminal antibody against CSB reveals only the expected major band for full length CSB(filled arrowhead) in MRC5 (SV40-immortalized human fetal lung fibroblast), E61ANd(GM00739B fibroblasts from compound heterozygote CS1AN, immortalized by SV40 and rescued by wt CSB cDNA [1]), Saos-2 (human osteosarcoma) and HT1080 (human fibrosarcoma); full length CSB is not seen in CS1AN/SV (the SV40-immortalized but unrescued parent of E61ANd). The >250 kDa species (labeled X) is seen only with Cterminal antibody and correlates with the abundance of full length CSB, but is also weakly expressed in the CS1AN/SV line lacking full length CSB; X may be a modified form of full length CSB, and weak expression in CS1AN/SV may indicate that one or both of the nonsense mutations in this compound heterozygote are leaky [2]. (B) Western blot using an N-terminal antibody against CSB reveals both full length CSB (filled arrowhead) and a second major band corresponding to the fusion protein (hollow arrowhead) in MRC5, Saos-2, and HT1080. We were also able to detect the fusion protein, but not full-length CSB, in SV40-immortalized primary fibroblasts from CSB patient CS1BE (derived from GM01629; data not shown). In agreement with our data, a band corresponding to the CSB-PGBD3 fusion protein was previously seen in the normal SV40-transformed WI38VA13 line [3] and in normal hTERT-immortalized BJ1 fibroblasts [4]. Although present in our CS1AN/hTERT line (Fig. 3), the fusion protein appears to be absent in the CS1AN/SV line (see also [3],[4]) and its derivatives, suggesting that SV40 immortalization may suppress the fusion protein. (C) Western blots using an N-terminal CSB antibody reveal only the fusion protein (hollow arrowhead) and not fulllength CSB (filled arrowhead) in three different primary CSB cells. GM00739B is derived from patient CS1AN and is the parent of our hTERT-immortalized derivatives (CSB-wt and [file pgen.1000031.s010.tif]

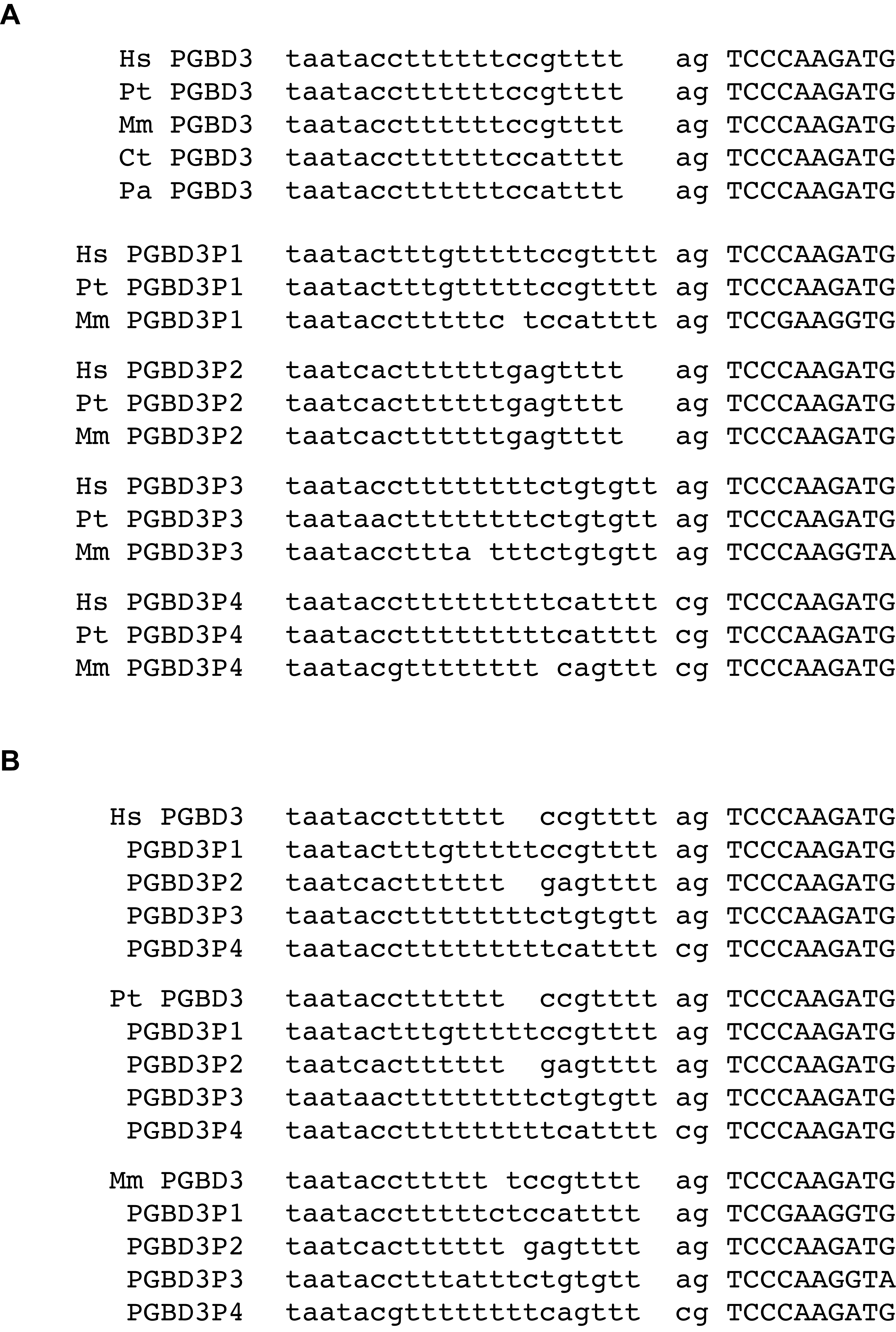

Supplement: Figure S4 — Conservation of the 3′ splice site in PGBD3. (A) Conserved 3′ splice site in the genomic sequence of PGBD3 and pseudogenes for human (Hs, Homo sapiens), chimpanzee (Pt, Pan troglodytes), Rhesus (Mm, Macaca mulatta), marmoset (Ct, Callithrix jacchus) and orangutan (Pa, Pongo abelli), organized by gene. Uppercase letters represent exon sequence; ATG at the 5′ end is the start codon of the PiggyBac ORF. Notably, the entire sequence shown is perfectly conserved in PGBD3 from all three species. (B) As for (A), organized by species to show the variations between PGBD3 and its pseudogenes. (1.47 MB TIF) [file pgen.1000031.s011.tif]

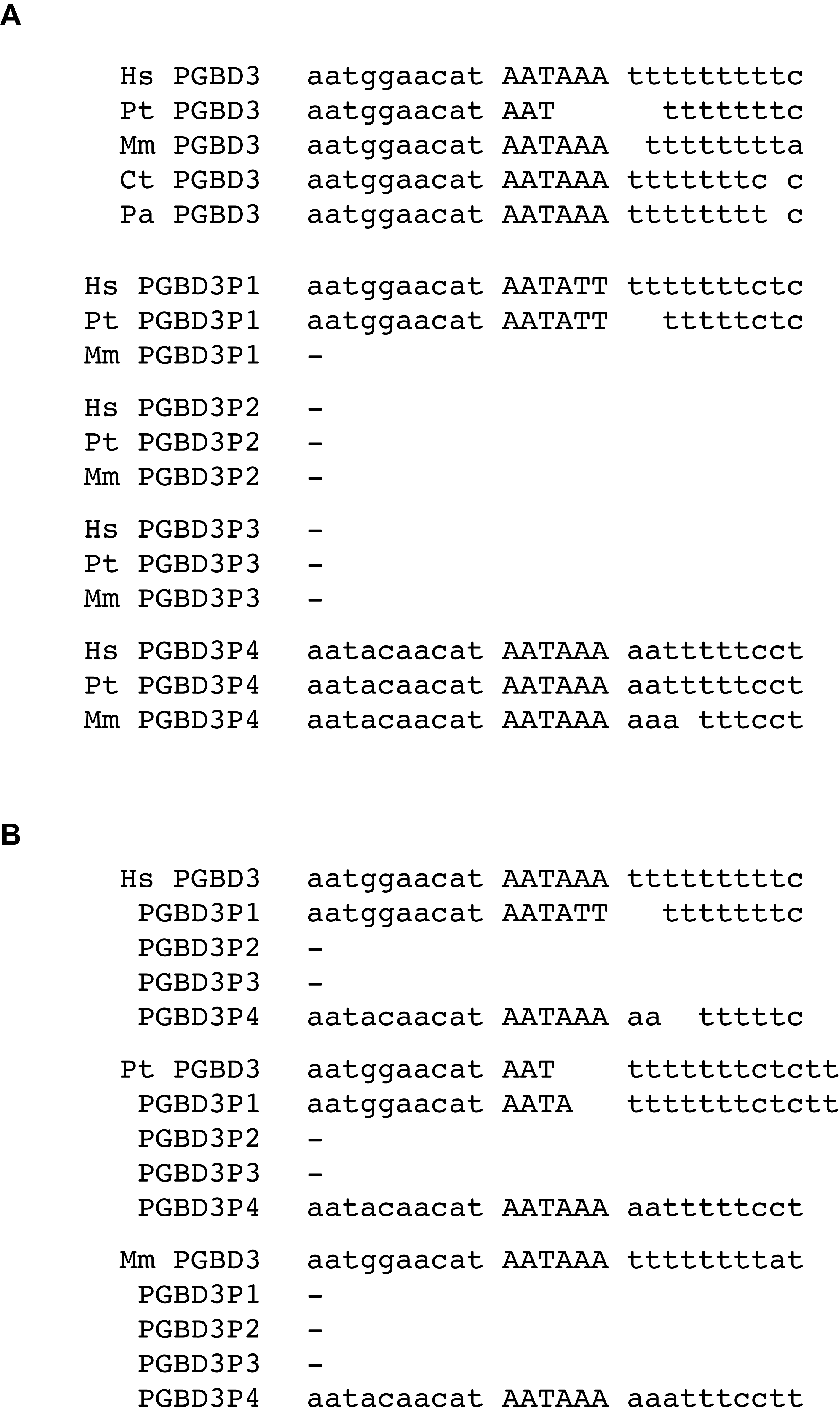

Supplement: Figure S5 — Conservation of the polyadenylation signal in PGBD3. (A) Conserved polyadenylation signals in the genomic sequence of PGBD3 and pseudogenes for human (Hs), chimpanzee (Pt), Rhesus (Mm), marmoset (Ct) and orangutan (Pa), organized by gene. Uppercase letters represent the AAUAAA motif. Dashes indicate pseudogenes for which the 3′ end (including the polyadenylation site) is no longer present. (B) As for (A), organized by species to show the variations between PGBD3 and its pseudogenes. (1.17 MB TIF) [file pgen.1000031.s012.tif]

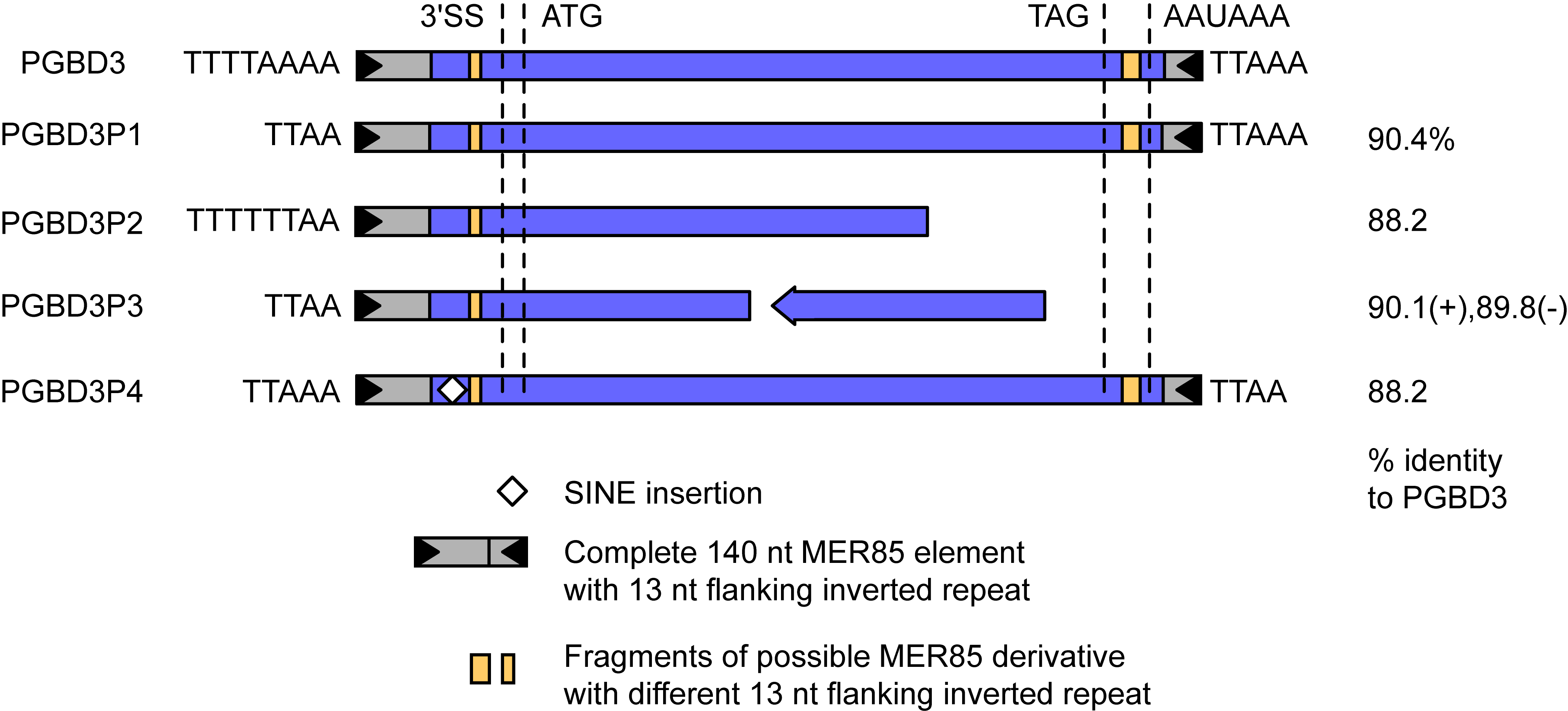

Supplement: Figure S6 — Schematic of PGBD3 element and the relationship with PGBD3 pseudogenes. The 5′ and 3′ ends of PGBD3 correspond to the 5′ arm (100 nt) and 3′ arm (40 nt) of a MER85 element (140 nt). The 13 nt inverted repeats of MER85 define the boundaries of the element, which is flanked by a TTAA target site duplication. Only two pseudogenes span the entire element, but all pseudogenes with intact ends share the same flanking features as PGBD3, and exhibit no homology to PGBD3 beyond the TTAA duplications. The 3′ end of PGBD3P3 is inverted, but the inverted sequence is as similar to PGBD3 as the remainder. An AluSx SINE has inserted into PGBD3P4 between the MER85 element and the 3′ SS. Also note two fragments (yellow) that appear to be derivatives of the left end of MER85, spanning 14 and 25 nt. The fragments each include the 5 innermost nt of the MER85 inverted repeat, but also share 8 further nt of unique flanking inverted repeat. Chimpanzee and Rhesus PGBD3 are structurally identical to human PGBD3, including all of the elements displayed. The chimpanzee pseudogenes are essentially identical to their human homologs. The Rhesus pseudogenes are more highly degraded, particularly at the 3′ end. (0.61 MB TIF) [file pgen.1000031.s013.tif]

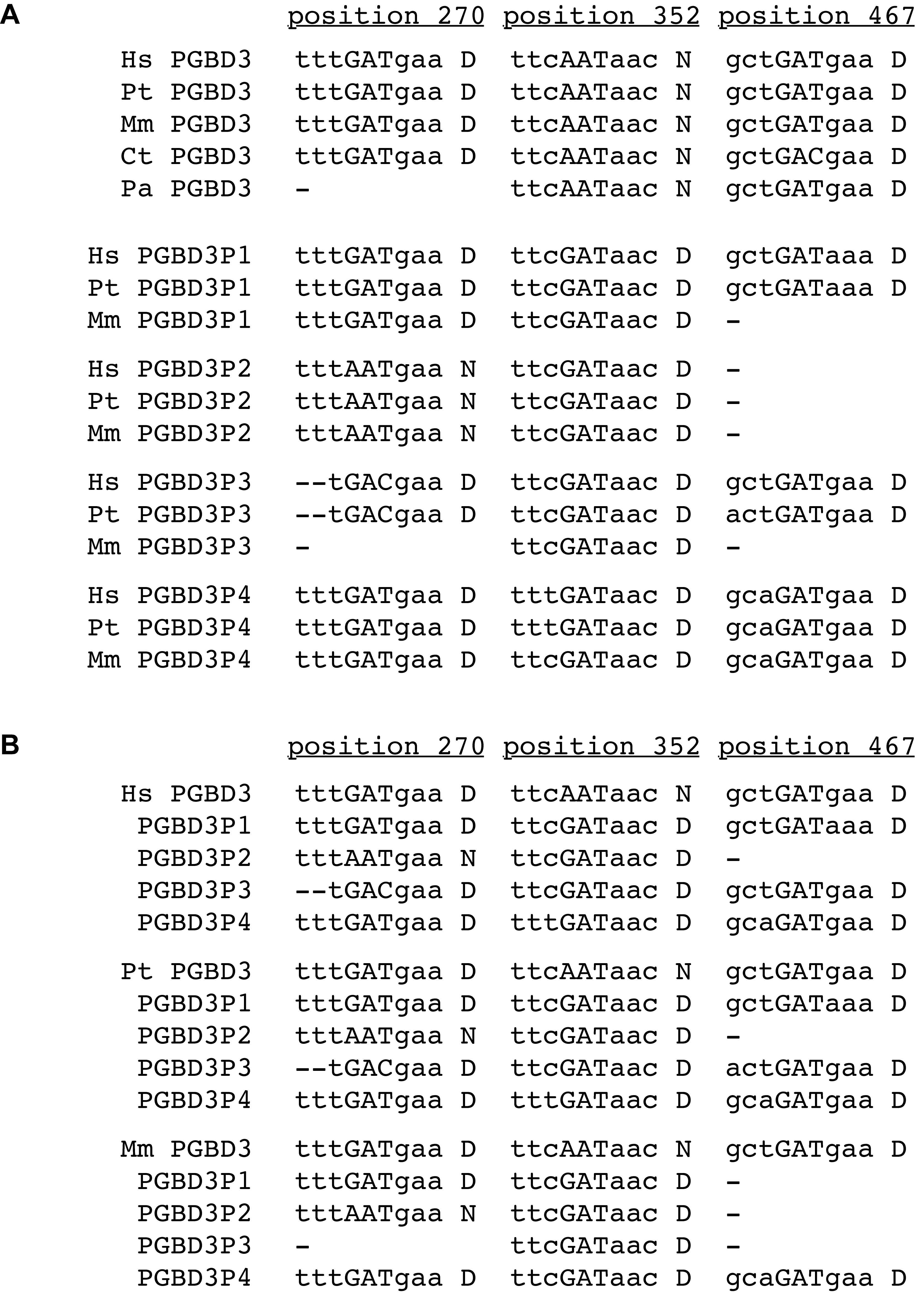

Supplement: Figure S7 — The putative catalytic motif is DDD in all pseudogenes, but DND in all PGBD3s. PiggyBac transposases share a DDD motif [1] that may be analogous to the metalcoordinating DDE motif common to other transposase families [2]. The most likely candidates for this motif in human PGBD3 are D270, N352 and D467. N352 is the result of a G to A mutation that occurred after generation of the four pseudogenes; all pseudogenes encode D at this position. (A) Genomic sequence of the three putative catalytic residues of PGBD3 and pseudogenes for human (Hs), chimpanzee (Pt), Rhesus (Mm), marmoset (Ct) and orangutan (Pa) organized by gene. Uppercase letters are the codons for the three residues. Dashes indicate pseudogenes for which the homologous sequence is no longer present; in addition, a 49 nt gap in the genome sequence of orangutan obscures D270. (B) As for (A), organized by species to show the variations between PGBD3 and its pseudogenes. (1.30 MB TIF) [file pgen.1000031.s014.tif]

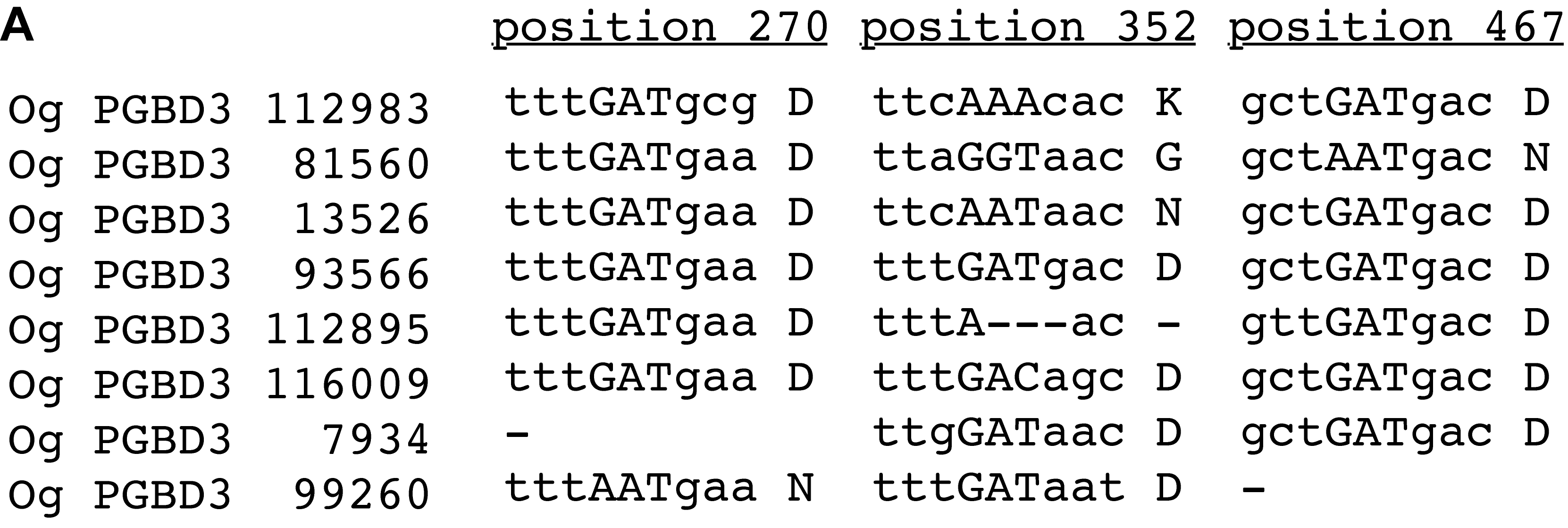

Supplement: Figure S8 — Consensus sequence of putative catalytic motif is DDD in galago PGBD3-related sequences. Six of seven sequences encode D at position 270, and six of seven encode D at 467. At position 352, four encode D while three encode K, G or N. Numbers designtating the PGBD3-like sequences represent the galago (Otolemur garnetti, Og) genomic contig on which the sequence is located. (0.36 MB TIF) [file pgen.1000031.s015.tif]
